# Supplementary material for: BALLI: Bartlett-adjusted likelihood-based linear model approach for identifying differentially expressed genes with RNA-seq data
Source: BMC Genomics. 2019 Jul 2;20:540. doi: 10.1186/s12864-019-5851-6 (PMC6604381; doi:10.1186/s12864-019-5851-6)
Supplement: Supplementary file 1 — The forms of Cg's components depending on the structure of Vg. (DOCX 17 kb) [file 12864_2019_5851_MOESM1_ESM.docx]

**Additional file 1**

In general cases, when $\psi_{g}$ and $\sigma_{g1}^{2},\ldots,\sigma_{gM}^{2}$ are unknown, and the latter is assumed to not be the same, if we let

$$V_{g}\mathbf{=}\psi_{g}\boldsymbol{\Sigma}_{g,\mathbf{b}}+\boldsymbol{\Sigma}_{g,\mathbf{e}}=\psi_{g}\Sigma_{g,\mathbf{b}}+\sum_{m=1}^{M} \sigma_{gm}^{2}\mathbf{I}_{\boldsymbol{n}_{\boldsymbol{m}}}=\sum_{j=1}^{M+1} \omega_{gj}Q_{gj},$$

where $Q_{g1}=\Sigma_{g,\mathbf{b}}$ and $Q_{gj}=\mathbf{I}_{\boldsymbol{n}_{\boldsymbol{j-1}}} \left( j=2,\ldots,M+1 \right)$, the Bartlett correction factor is derived as

$$C_{g}=tr\left( {\boldsymbol{D}_{\boldsymbol{g}}}^{-1}\left( -\frac{1}{2}\boldsymbol{M}_{\boldsymbol{g}}+\frac{1}{4}\boldsymbol{P}_{\boldsymbol{g}}-\frac{1}{2}\boldsymbol{\nu}_{\boldsymbol{g}}{\boldsymbol{\tau}_{\boldsymbol{g}}}^{t} \right) \right),$$

where $D_{g}$, $M_{g}$, and $P_{g}$ are $m\times m$ dimensional matrices, and if we let $\dot{X}_{l}^{'}=Z{(Z^{t}{V_{g}}^{-1}Z)}^{-1}Z^{t}{V_{g}}^{-1}Q_{gl}{V_{g}}^{-1}X^{'}$, they are expressed by

$\boldsymbol{D}_{\boldsymbol{g}}=\left\{ -\frac{1}{2}\mathrm{tr}({V_{g}}^{-1}Q_{gk}{V_{g}}^{-1}Q_{gl}) \right\}$,

$\boldsymbol{M}_{\boldsymbol{g}}=\left\{ 2\mathrm{tr}\left( \left( {X^{'}}^{t}{V_{g}}^{-1}X^{'} \right)^{-1}({X^{'}}^{t}{V_{g}}^{-1}Q_{gl}{V_{g}}^{-1}Q_{gk}{V_{g}}^{-1}X^{'}-{\dot{X}_{l}^{'}}^{t}{V_{g}}^{-1}Q_{gk}{V_{g}}^{-1}X^{'}) \right) \right\}$,

$\mathbf{P}_{\boldsymbol{g}}=\left\{ \mathrm{tr}\left( {X^{'}}^{t}{V_{g}}^{-1}Q_{gk}{V_{g}}^{-1}X^{'}{({X^{'}}^{t}{V_{g}}^{-1}X^{'})}^{-1}{X^{'}}^{t}{V_{g}}^{-1}Q_{gl}{V_{g}}^{-1}X^{'}{({X^{'}}^{t}{V_{g}}^{-1}X^{'})}^{-1} \right) \right\}$.

$\boldsymbol{\nu}_{\boldsymbol{g}}$ and $\boldsymbol{\tau}_{\boldsymbol{g}}$ are (M+1)-dimensional column vectors, and their *k*th elements are, respectively,

$\mathrm{tr}\left( -\left( Z^{t}{V_{g}}^{-1}Z \right)^{-1}\left( Z^{t}{V_{g}}^{-1}Q_{gk}{V_{g}}^{-1}Z \right) \right)$ and $\mathrm{tr}\left( -{({X^{'}}^{t}{V_{g}}^{-1}X^{'})}^{-1}\left( {X^{'}}^{t}{V_{g}}^{-1}Q_{gk}{V_{g}}^{-1}X^{'} \right) \right)$.

Moreover, this approach can be easily applied with some modification of $Q_{gj}$ to special cases, such as when $\psi_{g}=1$ and $\sigma_{g1}^{2},\ldots,\sigma_{gM}^{2}$ are unknown and not the same, $\psi_{g}$ and $\sigma_{g1}^{2}=\ldots=\sigma_{gM}^{2}$ are unknown and $\psi_{g}$ is unknown, and $\sigma_{g1}^{2}=\ldots=\sigma_{gM}^{2}=0$. $Q_{gj}$ values for various $V_{g}$ values are summarized as follows:

| Models | $V_{g}$ and $Q_{gk}$ |
| --- | --- |
| 1. $\psi_{g}=1$,   $\sigma_{g1}^{2}=\ldots=\sigma_{gM}^{2}$ | $V_{g}\mathbf{=}\Sigma_{g,\mathbf{b}}+\omega_{g1}Q_{g1},$ $Q_{g1}=\mathbf{I}$ |
| 1. $\psi_{g}$: unknown, $\sigma_{g1}^{2}, \ldots, \sigma_{gM}^{2}$: not same | $V_{g}\mathbf{=}\sum_{j=1}^{M+1} \omega_{gj}Q_{gj},$  $Q_{g1}=\Sigma_{g,\mathbf{b}}$ and $Q_{gj}=\mathbf{I}_{\boldsymbol{n}_{\boldsymbol{j-1}}} \left( j=2,\ldots,M+1 \right)$ |
| 1. $\psi_{g}=1$,   $\sigma_{g1}^{2}, \ldots, \sigma_{gM}^{2}$: not same | $V_{g}=\Sigma_{g,\mathbf{b}}+\sum_{j=1}^{M} \omega_{gj}Q_{gj},$  $Q_{gj}=\mathbf{I}_{\boldsymbol{n}_{\boldsymbol{j}}} (j=1,\ldots M)$ |
| 1. $\psi_{g}$: unknown,   $\sigma_{g1}^{2}=\ldots=\sigma_{gM}^{2}$ | $V_{g}\mathbf{=}\sum_{j=1}^{2} \omega_{gj}Q_{gj}, Q_{g1}=\Sigma_{g,\mathbf{b}}$ and $Q_{g2}=\mathbf{I}$ |
| 1. $\psi_{g}$: unknown,   $\sigma_{g1}^{2}=\ldots=\sigma_{gM}^{2}=0$ | $V_{g}\mathbf{=}\omega_{g1}Q_{g1},$ $Q_{g1}=\Sigma_{g,\mathbf{b}}$ |
