# Supplementary material for: BALLI: Bartlett-adjusted likelihood-based linear model approach for identifying differentially expressed genes with RNA-seq data
Source: BMC Genomics. 2019 Jul 2;20:540. doi: 10.1186/s12864-019-5851-6 (PMC6604381; doi:10.1186/s12864-019-5851-6)
Supplement: Supplementary file 2 — Steps of generating simulation data. (DOCX 18 kb) [file 12864_2019_5851_MOESM2_ESM.docx]

**Additional file 2**

**Simulation studies with Nigerian RNA-seq data**

Simulation data were generated by modifying Nigerian RNA-seq data. We considered various sample sizes (N = 4, 6, 8, 12, 16, 20, 24, 28, 40, 64, and 68). We assumed that there were two groups (cases and controls), and the sample sizes of both groups were assumed to be the same. Below are the details for the null dataset generation.

1. Remove genes whose read counts across all samples were less than a tenth of the number of samples.
2. For all simulation datasets with sample sizes of 4, 6, 8, 12, 16, 20, 24, 28, 40, 64, and 68, we assumed that numbers of males and females are same. For instance, if the same size is 4, the numbers of males and females are 2. We used the *sample* function in R to randomly select the certain numbers of males and females
3. We repeated this process 50 times for each sample size and used the data to estimate the empirical type-1 error.

Below are the details for how we generated datasets with artificial DEGs.

1. A certain number of genes was randomly selected from the null dataset. In this process, we first set the seed using *set.seed(i),* where *i* represents the iteration number; then, the first few genes in order were selected according to *sample(1:ng),* where ng represents the number of genes in each null dataset. We randomly chose genes from each dataset to be artificial DEGs.
2. For the first half of the selected DEGs, we added a certain value, δ, to the read counts of samples in one group, and for the other half, δ was added to the read counts of samples in the other group. If we denoted the sample standard deviation of the read counts of a gene by σ, we considered δ = 0.5σ or 1σ.
3. We generated 50 replicates for each scenario, and empirical powers and precisions were estimated with all DEGs.

**Simulation studies with Holstein RNA-seq data**

Null simulation dataset derived from Holstein data were generated by the same manner of generating those from Nigerian data. Low and high milk yielding group samples were selected according to first few order of *sample(1:12)* and *sample(1:9)* after being seeded using *set.seed(*$100\times i$*)* and *set.seed(*$1000\times i$*)*, respectively, where *i* represents iteration number of generation. Each simulation dataset with sample size of 4, 6, 8, 12, 16 and 20 include 2 low and 2 high, 4 low and 2 high, 4 low and 4 high, 6 low and 6 high, 8 low and 8 high and 12 low and 8 high milk yielding group samples, respectively. Procedures to generate dataset including artificial DEGs were also same as Nigerian’s

**Simulation studies with randomly generated RNA-seq data**

For the second scenario in simulation studies, we generated RNA-seq data from the negative binomial distribution. We assumed that there were two groups (case versus control), and *N* was assumed to be 4, 6, 8, 12, 16, 20, 24, 28, 40, 64, or 68. We assumed that the number of genes was the same as that in the Nigerian people’s RNA-seq data, and if we let the mean and variance of $r_{gi}$ be $\mu_{g}$ and $\phi_{g}$ respectively, $r_{gi}$ was generated from $NB(a_{gi},{(0.2+a_{gi}^{-1/2})}^{2}\delta_{g})$. Here, $\delta_{g}$ was generated from the inverse chi-square distribution with 40 degrees of freedom. $a_{gi}$ was obtained as the product of $\mu_{g}$ and $c_{i}$, where $\mu_{g}$ was generated from the negative binomial distributions with the mean and overdispersion parameters based on their estimates from the corresponding genes in the Nigerian people’s RNA-seq data. *c_i_* was incorporated to evaluate the effects of library size variation. N/2 number of *c_i_* was independently generated from $U\left( 2-u,u \right)$, where $u\in\{0.2, 0.4, 0.6, 0.8, 1\}$. Each *c_i_* was used for a pair of a case and control, which made the total read depths for cases and controls the same. Notably, *u* = 1 led to the same read depth among subjects, and $u$ = 0.2 generated the largest variation of read depths among subjects. We assumed that 20% of genes were DEGs, and empirical type-1 errors and powers were estimated by adding δ to the simulated RNA-seq data, as was performed for simulation studies with real RNA-seq data. All procedures were repeated 20 times.
