# Supplementary material for: BALLI: Bartlett-adjusted likelihood-based linear model approach for identifying differentially expressed genes with RNA-seq data
Source: BMC Genomics. 2019 Jul 2;20:540. doi: 10.1186/s12864-019-5851-6 (PMC6604381; doi:10.1186/s12864-019-5851-6)
Supplement: Supplementary file 3 — Estimated type-1 error rates with simulation data for N = 4, 6, 8, 28, 40, 64, and 68 based on Nigerian people’s data. (DOCX 21 kb) [file 12864_2019_5851_MOESM3_ESM.docx]

**Additional file 3**

Estimated type-1 error rates with simulation data based on Nigerian people’s data. Estimated type-1 error rates by BALLI, DESeq2, edgeR, LLI, and voom and their 95% confidence levels were estimated for $N=4, 6, 8, 28, 40, 64, and 68$. The type-1 error rates are marked by bold font if their 95% confidence levels include or lower than the nominal significant level $\alpha$.

| $\alpha$ | *N* = 4 | | | | | *N* = 6 | | | | |
| --- | --- | --- | --- | --- | --- | --- | --- | --- | --- | --- |
|  | BALLI | DESeq2 | edgeR | LLI | voom | BALLI | DESeq2 | edgeR | LLI | voom |
| 0.1 | 0.16937  (0.15499,0.18374) | **0.06929**  **(0.05899,0.07958)** | **0.10811**  **(0.09553,0.12069)** | 0.26703  (0.24928,0.28478) | **0.10782**  **(0.09318,0.12245)** | 0.11539  (0.10539,0.12539) | **0.07197**  **(0.06345,0.08049)** | **0.10039**  **(0.09164,0.10915)** | 0.18595  (0.17327,0.19864) | **0.09945**  **(0.08791,0.11099)** |
| 0.05 | 0.09899  (0.08863,0.10934) | **0.04205**  **(0.03466,0.04945)** | 0.06373  (0.05476,0.07269) | 0.19250  (0.17706,0.20793) | **0.05699**  **(0.04692,0.06707)** | 0.06219  (0.05548,0.06891) | **0.04082**  **(0.03499,0.04666)** | **0.05582**  **(0.04977,0.06188)** | 0.11344  (0.10346,0.12342) | **0.05018**  **(0.04283,0.05754)** |
| 0.01 | 0.01981  (0.01638,0.02323) | 0.01634  (0.01271,0.01998) | 0.02307  (0.01887,0.02727) | 0.08002  (0.07070,0.08934) | 0.01419  (0.01054,0.01784) | 0.01475  (0.01257,0.01694) | 0.01343  (0.01085,0.01601) | 0.01788  (0.01515,0.02061) | 0.03956  (0.03472,0.04440) | **0.01042**  **(0.00828,0.01256)** |
| 0.005 | 0.00923  (0.00740,0.01105) | 0.01160  (0.00884,0.01436) | 0.01608  (0.01291,0.01926) | 0.05346  (0.04617,0.06075) | 0.00787  (0.00552,0.01022) | 0.00703  (0.00579,0.00828) | 0.00890  (0.00710,0.01069) | 0.01202  (0.01004,0.01401) | 0.02499  (0.02163,0.02836) | **0.00538**  **(0.00415,0.00660)** |
| $\alpha$ | *N* = 8 | | | | | *N* = 28 | | | | |
|  | BALLI | DESeq2 | edgeR | LLI | voom | BALLI | DESeq2 | edgeR | LLI | voom |
| 0.1 | **0.10385**  **(0.09510,0.11259)** | **0.08122**  **(0.07284,0.08960)** | **0.10522**  **(0.09740,0.11304)** | 0.15233  (0.14161,0.16304) | **0.10435**  **(0.09368,0.11501)** | **0.09211**  **(0.08249,0.10173)** | **0.09696**  **(0.08607,0.10785)** | 0.11723  (0.10699,0.12747) | **0.10656**  **(0.09606,0.11707)** | **0.10083**  **(0.08961,0.11206)** |
| 0.05 | **0.05199**  **(0.04665,0.05733)** | **0.04504**  **(0.03932,0.05076)** | 0.05681  (0.05127,0.06234) | 0.09115  (0.08308,0.09922) | **0.05119**  **(0.04481,0.05757)** | **0.04419**  **(0.03806,0.05032)** | **0.05167**  **(0.04419,0.05915)** | 0.06515  (0.05818,0.07212) | **0.05368**  **(0.04672,0.06065)** | **0.04995**  **(0.04270,0.05720)** |
| 0.01 | 0.01159  (0.01018,0.01300) | 0.01362  (0.01130,0.01594) | 0.01752  (0.01502,0.02002) | 0.02632  (0.02331,0.02933) | **0.01035**  **(0.00851,0.01219)** | **0.00787**  **(0.00617,0.00957)** | 0.01308  (0.01044,0.01573) | 0.01599  (0.01363,0.01835) | **0.01095**  **(0.00873,0.01317)** | **0.00958**  **(0.00761,0.01154)** |
| 0.005 | 0.00615  (0.00534,0.00696) | 0.00897  (0.00729,0.01064) | 0.01140  (0.00952,0.01329) | 0.01589  (0.01395,0.01784) | **0.00543**  **(0.00434,0.00652)** | **0.00367**  **(0.00280,0.00453)** | 0.00749  (0.00583,0.00915) | 0.00902  (0.00754,0.01050) | **0.00546**  **(0.00421,0.00670)** | **0.00469**  **(0.00364,0.00575)** |
| $\alpha$ | *N* = 40 | | | | | *N* = 64 | | | | |
|  | BALLI | DESeq2 | edgeR | LLI | voom | BALLI | DESeq2 | edgeR | LLI | voom |
| 0.1 | **0.08709**  **(0.07736,0.09682)** | **0.09254**  **(0.08224,0.10285)** | **0.10950**  **(0.09983,0.11916)** | **0.09657**  **(0.08636,0.10678)** | **0.09652**  **(0.08514,0.10789)** | **0.09429**  **(0.08245,0.10612)** | **0.10312**  **(0.09119,0.11505)** | 0.11989  (0.10819,0.13160) | **0.10032**  **(0.08817,0.11248)** | **0.10874**  **(0.09457,0.12291)** |
| 0.05 | **0.04173**  **(0.03523,0.04822)** | **0.04873**  **(0.04134,0.05612)** | 0.05999  (0.05334,0.06665) | **0.04796**  **(0.04091,0.05502)** | **0.04739**  **(0.03988,0.05491)** | **0.04699**  **(0.03850,0.05547)** | **0.05652**  **(0.04759,0.06546)** | 0.06578  (0.05727,0.07429) | **0.05109**  **(0.04224,0.05995)** | **0.05620**  **(0.04628,0.06611)** |
| 0.01 | **0.00743**  **(0.00542,0.00944)** | **0.01212**  **(0.00916,0.01509)** | 0.01518  (0.01261,0.01775) | **0.00946**  **(0.00702,0.01190)** | **0.00907**  **(0.00674,0.01140)** | **0.00954**  **(0.00615,0.01293)** | 0.01487  (0.01087,0.01887) | 0.01769  (0.01413,0.02126) | **0.01100**  **(0.00731,0.01468)** | **0.01222**  **(0.00863,0.01581)** |
| 0.005 | **0.00357**  **(0.00237,0.00476)** | **0.00676**  **(0.00487,0.00864)** | 0.00841  (0.00678,0.01003) | **0.00475**  **(0.00329,0.00621)** | **0.00431**  **(0.00300,0.00562)** | **0.00499**  **(0.00275,0.00723)** | 0.00848  (0.00569,0.01127) | 0.01033  (0.00789,0.01277) | **0.00575**  **(0.00331,0.00819)** | **0.00647**  **(0.00419,0.00876)** |
| $\alpha$ | *N* = 68 | | | | |  |  |  |  |  |
|  | BALLI | DESeq2 | edgeR | LLI | voom |  |  |  |  |  |
| 0.1 | **0.09276**  **(0.08110,0.10443)** | **0.10257**  **(0.09069,0.11446)** | 0.11950  (0.10789,0.13110) | **0.09837**  **(0.08638,0.11035)** | **0.10502**  **(0.09123,0.11881)** |  |  |  |  |  |
| 0.05 | **0.04608**  **(0.03776,0.05441)** | **0.05594**  **(0.04705,0.06482)** | 0.06542  (0.05693,0.07390) | **0.04991**  **(0.04118,0.05863)** | **0.05397**  **(0.04425,0.06368)** |  |  |  |  |  |
| 0.01 | **0.00960**  **(0.00632,0.01289)** | 0.01457  (0.01074,0.01841) | 0.01765  (0.01416,0.02114) | **0.01092**  **(0.00734,0.01450)** | **0.01165**  **(0.00821,0.01509)** |  |  |  |  |  |
| 0.005 | **0.00500**  **(0.00291,0.00708)** | 0.00854  (0.00590,0.01119) | 0.01035  (0.00802,0.01268) | **0.00579**  **(0.00347,0.00810)** | **0.00616**  **(0.00399,0.00834)** |  |  |  |  |  |
