# Supplementary material for: BALLI: Bartlett-adjusted likelihood-based linear model approach for identifying differentially expressed genes with RNA-seq data
Source: BMC Genomics. 2019 Jul 2;20:540. doi: 10.1186/s12864-019-5851-6 (PMC6604381; doi:10.1186/s12864-019-5851-6)
Supplement: Supplementary file 4 — Estimated type-1 error rates with simulation data for N = 4, 6, 8, 12, 16 and 20 based on Holstein cow’s data. (DOCX 20 kb) [file 12864_2019_5851_MOESM4_ESM.docx]

**Additional file 4**

Estimated type-1 error rates with simulation data based on Holstein cow’s data. Estimated type-1 error rates by BALLI, DESeq2, edgeR, LLI, and voom and their 95% confidence levels were estimated for $N=4, 6, 8, 12, 16 and 20$. The type-1 error rates are marked by bold font if their 95% confidence levels include or lower than the nominal significant level $\alpha$.

| $\alpha$ | *N* = 4 | | | | | *N* = 6 | | | | |
| --- | --- | --- | --- | --- | --- | --- | --- | --- | --- | --- |
|  | BALLI | DESeq2 | edgeR | LLI | voom | BALLI | DESeq2 | edgeR | LLI | voom |
| 0.1 | 0.14714  (0.12776,0.16651) | **0.09061**  **(0.07379,0.10743)** | **0.09461**  **(0.07862,0.11060)** | 0.24915  (0.22363,0.27467) | **0.08975**  **(0.07131,0.10819)** | 0.13109  (0.10858,0.15359) | **0.11737**  **(0.09588,0.13887)** | **0.11803**  **(0.09864,0.13742)** | 0.20855  (0.18021,0.23688) | **0.11309**  **(0.09005,0.13613)** |
| 0.05 | 0.08720  (0.07390,0.10049) | **0.05578**  **(0.04353,0.06804)** | **0.05439**  **(0.04308,0.06569)** | 0.17271  (0.15132,0.19410) | **0.04573**  **(0.03363,0.05782)** | 0.07219  (0.05669,0.08768) | 0.07288  (0.05686,0.08891) | 0.06950  (0.05571,0.08328) | 0.13044  (0.10798,0.15290) | **0.05907**  **(0.04366,0.07448)** |
| 0.01 | 0.02364  (0.01887,0.02841) | 0.02224  (0.01628,0.02819) | 0.01945  (0.01408,0.02483) | 0.07710  (0.06488,0.08933) | **0.01057**  **(0.00653,0.01461)** | 0.01839  (0.01314,0.02365) | 0.02834  (0.02062,0.03607) | 0.02461  (0.01848,0.03074) | 0.04734  (0.03599,0.05868) | **0.01267**  **(0.00805,0.01728)** |
| 0.005 | 0.01280  (0.00988,0.01571) | 0.01587  (0.01131,0.02043) | 0.01335  (0.00931,0.01738) | 0.05448  (0.04514,0.06382) | **0.00577**  **(0.00332,0.00822)** | 0.01023  (0.00705,0.01342) | 0.01981  (0.01413,0.02550) | 0.01666  (0.01223,0.02109) | 0.03085  (0.02277,0.03893) | **0.00651**  **(0.00390,0.00912)** |
| $\alpha$ | *N* = 8 | | | | | *N* = 12 | | | | |
|  | BALLI | DESeq2 | edgeR | LLI | voom | BALLI | DESeq2 | edgeR | LLI | voom |
| 0.1 | **0.10706**  **(0.08263,0.13150)** | **0.10830**  **(0.08317,0.13342)** | **0.10841**  **(0.08602,0.13080)** | 0.15973  (0.13024,0.18923) | **0.10163**  **(0.07655,0.12670)** | **0.10227**  **(0.08210,0.12245)** | **0.11604**  **(0.09421,0.13787)** | **0.11568**  **(0.09680,0.13456)** | 0.13814  (0.11454,0.16174) | **0.10704**  **(0.08452,0.12956)** |
| 0.05 | **0.05578**  **(0.03919,0.07237)** | **0.06582**  **(0.04652,0.08513)** | **0.06278**  **(0.04605,0.07951)** | 0.09404  (0.07125,0.11684) | **0.05282**  **(0.03528,0.07037)** | **0.04944**  **(0.03701,0.06186)** | 0.06606  (0.05038,0.08174) | **0.06214**  **(0.04932,0.07496)** | 0.07497  (0.05837,0.09157) | **0.05358**  **(0.03896,0.06819)** |
| 0.01 | **0.01290**  **(0.00698,0.01882)** | 0.02425  (0.01421,0.03429) | 0.02080  (0.01278,0.02881) | 0.02866  (0.01799,0.03932) | **0.01172**  **(0.00560,0.01784)** | **0.00929**  **(0.00602,0.01255)** | 0.01926  (0.01304,0.02547) | 0.01598  (0.01132,0.02063) | 0.01840  (0.01257,0.02424) | **0.00961**  **(0.00588,0.01335)** |
| 0.005 | **0.00707**  **(0.00345,0.01069)** | 0.01636  (0.00894,0.02378) | 0.01381  (0.00789,0.01973) | 0.01758  (0.01010,0.02507) | **0.00625**  **(0.00247,0.01004)** | **0.00454**  **(0.00273,0.00635)** | 0.01159  (0.00757,0.01560) | 0.00925  (0.00627,0.01223) | 0.00987  (0.00642,0.01333) | **0.00435**  **(0.00241,0.00629)** |
| $\alpha$ | *N* = 16 | | | | | *N* = 20 | | | | |
|  | BALLI | DESeq2 | edgeR | LLI | voom | BALLI | DESeq2 | edgeR | LLI | voom |
| 0.1 | **0.10662**  **(0.08602,0.12722)** | 0.12416  (0.10274,0.14557) | 0.12531  (0.10620,0.14442) | 0.13546  (0.11251,0.15841) | **0.11144**  **(0.08961,0.13327)** | **0.09289**  **(0.07631,0.10947)** | **0.11345**  **(0.09618,0.13072)** | 0.11946  (0.10416,0.13477) | **0.11501**  **(0.09679,0.13324)** | **0.09780**  **(0.07953,0.11607)** |
| 0.05 | **0.05240**  **(0.03861,0.06619)** | 0.07301  (0.05665,0.08936) | 0.06997  (0.05586,0.08408) | 0.07218  (0.05548,0.08887) | **0.05627**  **(0.04130,0.07124)** | **0.04389**  **(0.03247,0.05532)** | 0.06346  (0.05087,0.07605) | 0.06464  (0.05397,0.07531) | **0.05791**  **(0.04476,0.07106)** | **0.04814**  **(0.03547,0.06080)** |
| 0.01 | **0.01020**  **(0.00578,0.01462)** | 0.02383  (0.01597,0.03169) | 0.02068  (0.01419,0.02716) | 0.01714  (0.01054,0.02375) | **0.01134**  **(0.00640,0.01628)** | **0.00842**  **(0.00415,0.01268)** | 0.01824  (0.01223,0.02425) | 0.01674  (0.01251,0.02097) | **0.01275**  **(0.00721,0.01830)** | **0.00922**  **(0.00482,0.01363)** |
| 0.005 | **0.00485**  **(0.00245,0.00725)** | 0.01525  (0.00968,0.02083) | 0.01296  (0.00829,0.01762) | 0.00913  (0.00509,0.01316) | **0.00558**  **(0.00282,0.00835)** | **0.00428**  **(0.00158,0.00697)** | 0.01120  (0.00686,0.01553) | 0.00988  (0.00704,0.01272) | **0.00693**  **(0.00316,0.01069)** | **0.00450**  **(0.00191,0.00710)** |
