# Supplementary material for: BALLI: Bartlett-adjusted likelihood-based linear model approach for identifying differentially expressed genes with RNA-seq data
Source: BMC Genomics. 2019 Jul 2;20:540. doi: 10.1186/s12864-019-5851-6 (PMC6604381; doi:10.1186/s12864-019-5851-6)

**Additional file 5**

Estimated powers and precisions with simulation data based on Holstein cow’s RNA-seq data. Statistical powers of BALLI, DESeq2, edgeR, LLI, and voom were estimated at FDR-adjusted 0.1 significance level when δ = 0.5σ or 1σ and $N=12, 16\mathrm{and}20$**.**


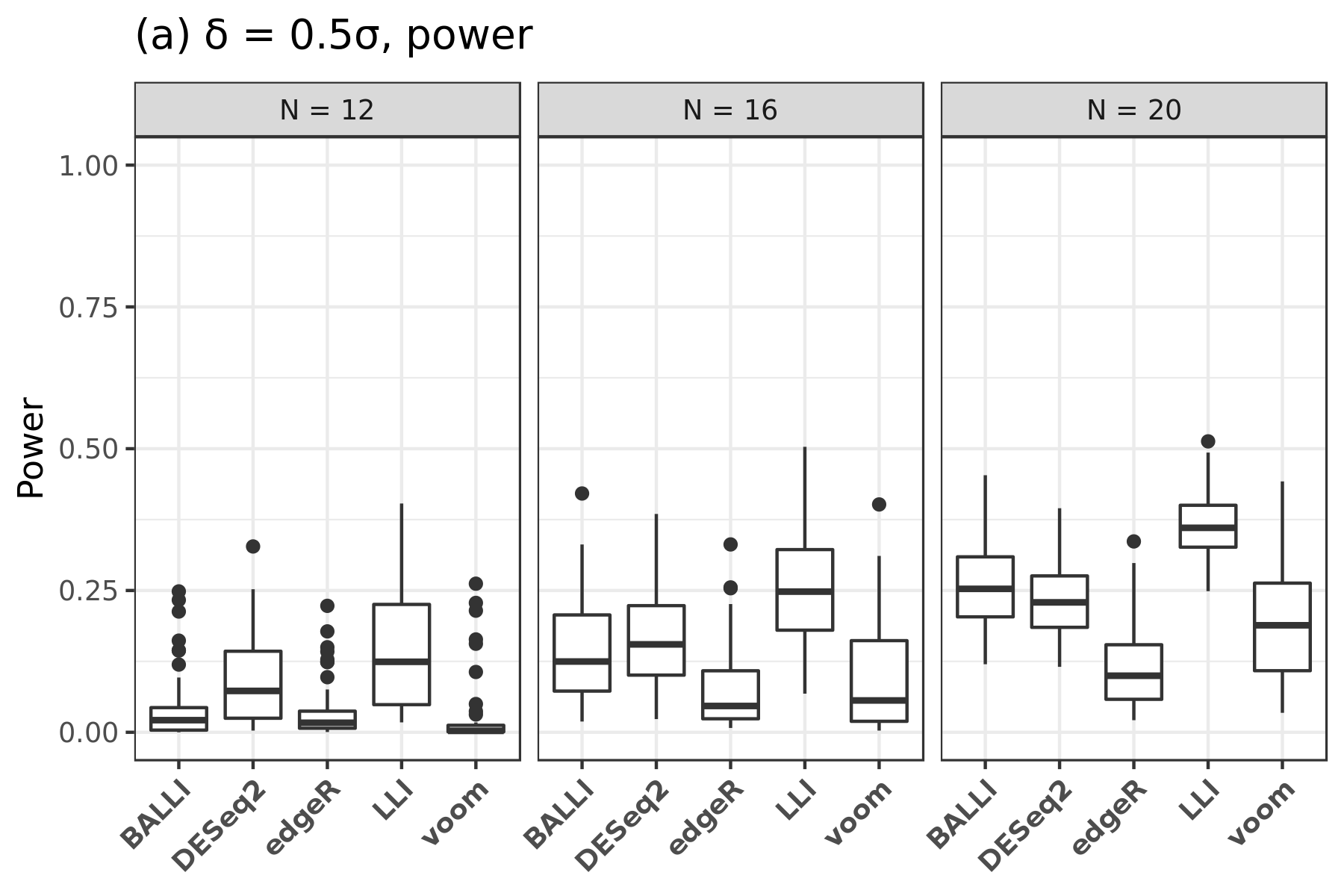

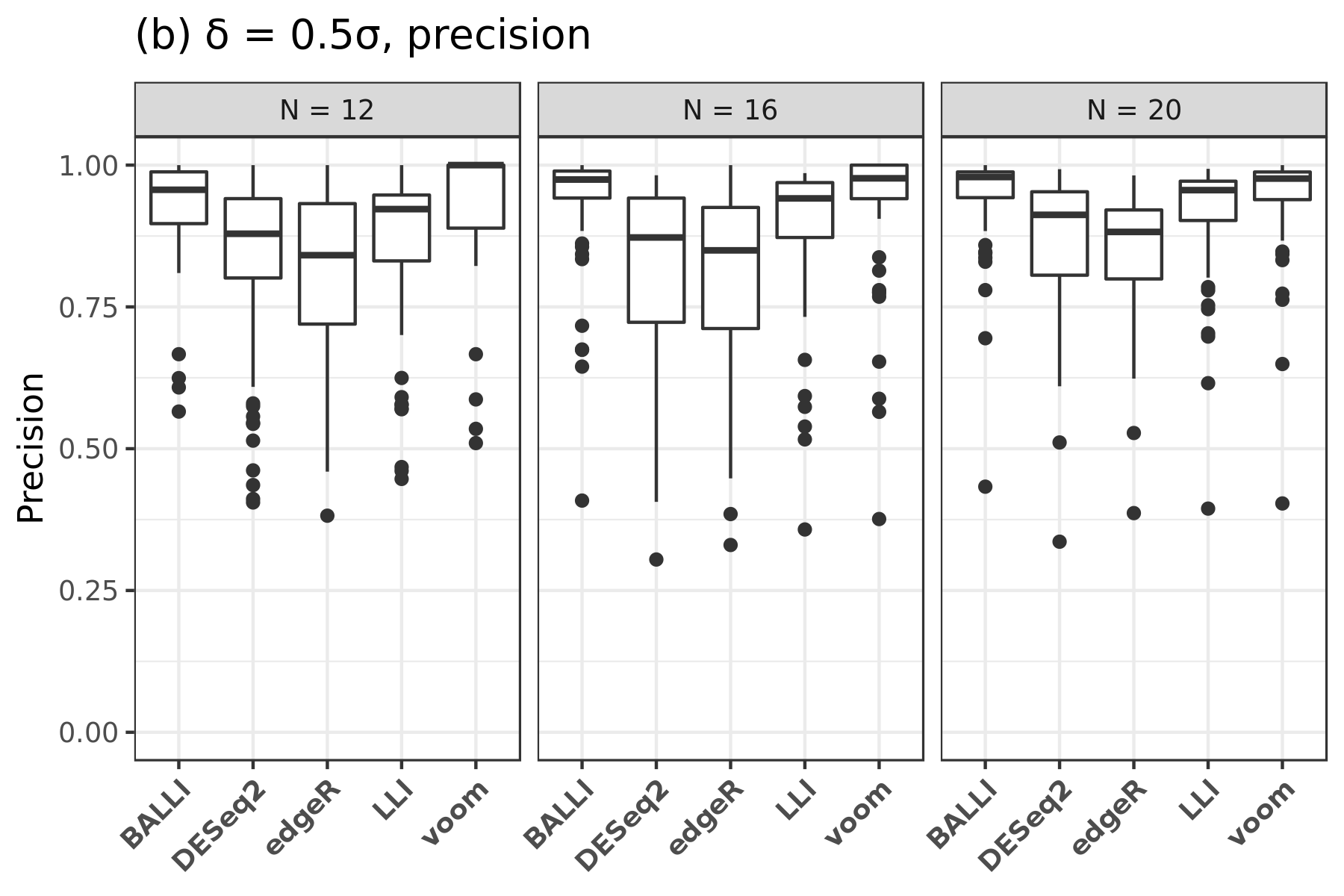


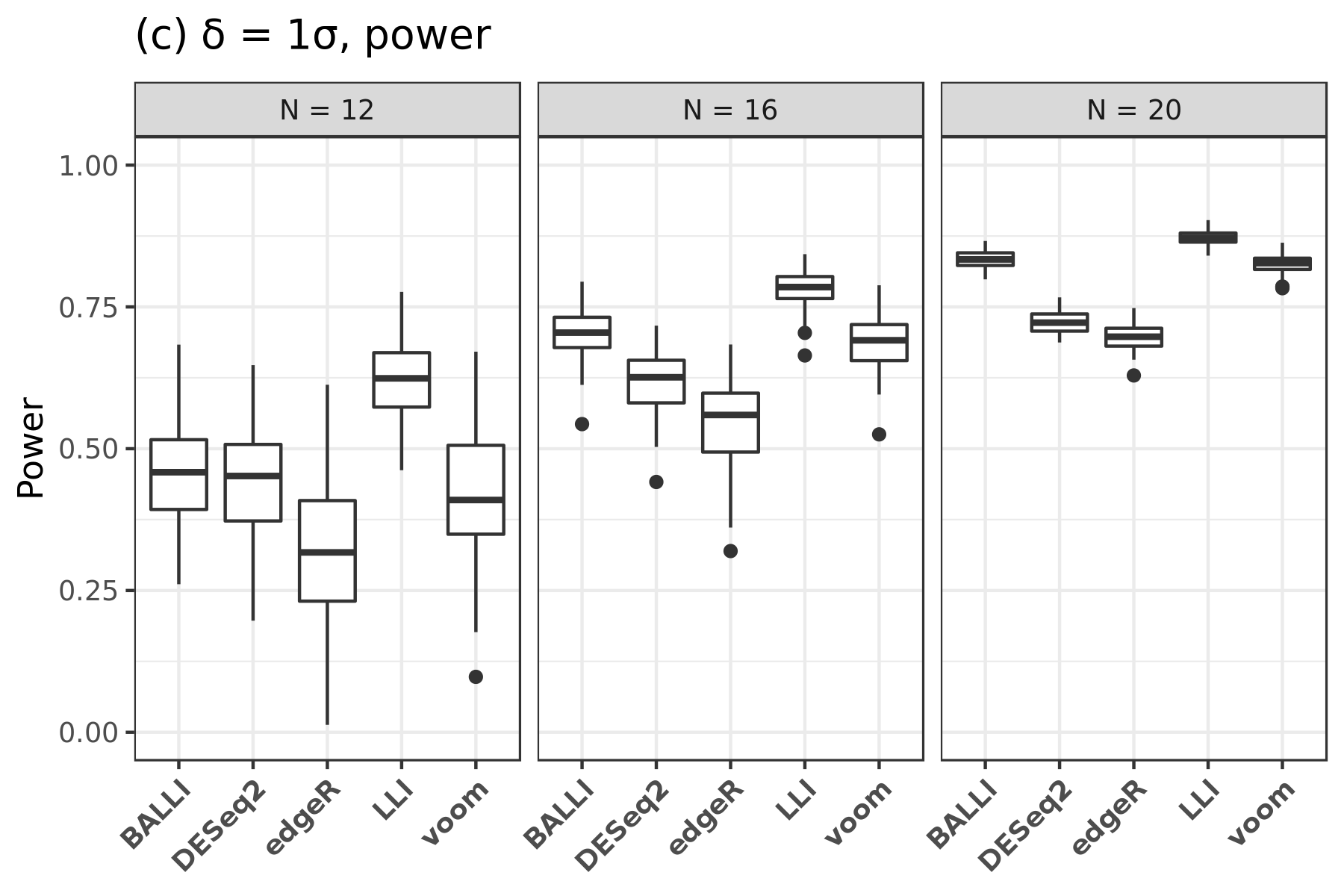

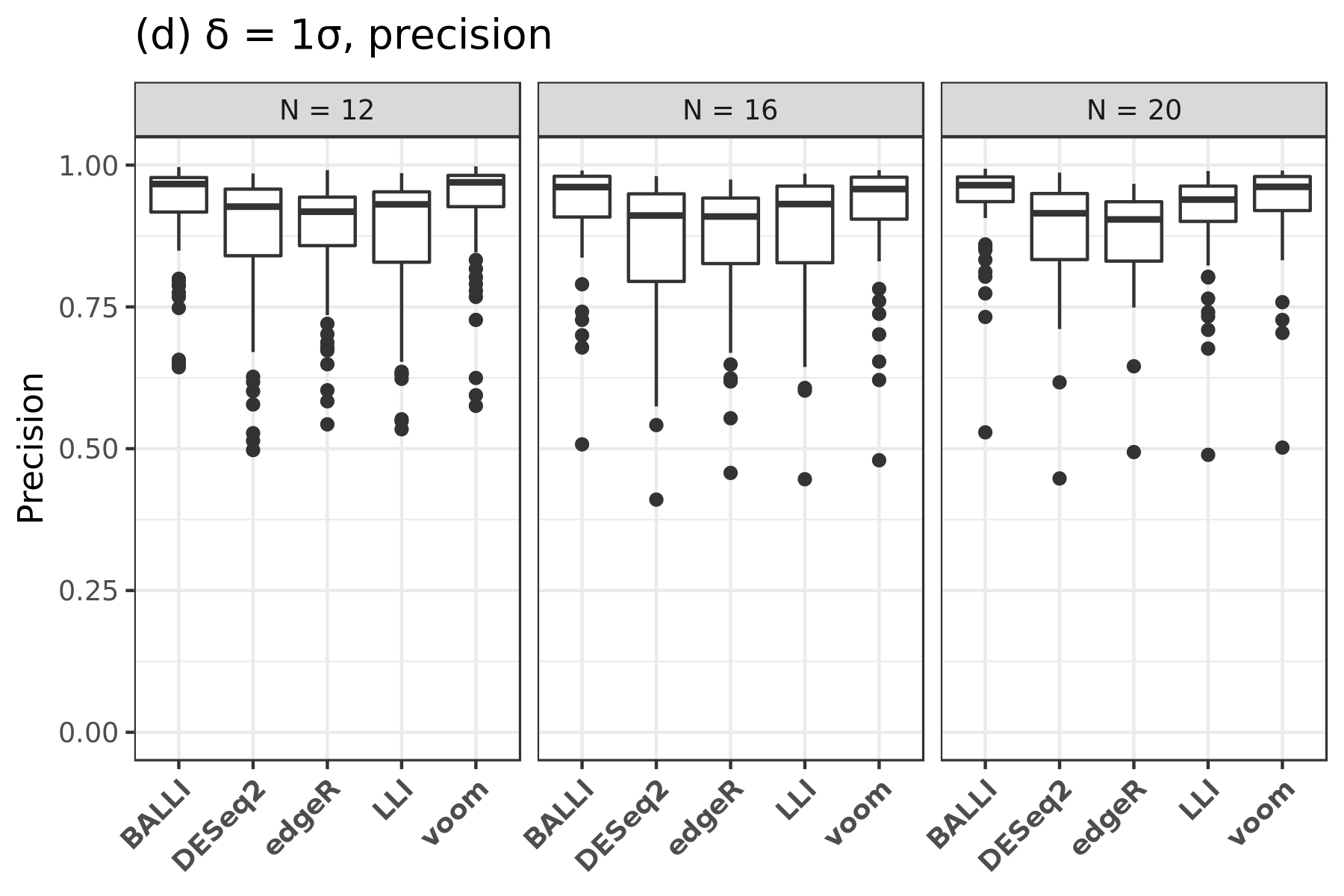

Supplement: Supplementary file 5 — Estimated powers and precisions with simulation data when δ = 0.5σ or 1σ and N = 12, 16 and 20 based on Holstein cow’s data. (DOCX 197 kb) [file 12864_2019_5851_MOESM5_ESM.docx]
