# Supplementary material for: BALLI: Bartlett-adjusted likelihood-based linear model approach for identifying differentially expressed genes with RNA-seq data
Source: BMC Genomics. 2019 Jul 2;20:540. doi: 10.1186/s12864-019-5851-6 (PMC6604381; doi:10.1186/s12864-019-5851-6)
Supplement: Supplementary file 6 — Estimated type-1 error rates with simulation data for N = 4, 6, 8, 28, 40, 64, and 68 based on simulated data from negative binomial distribution. (DOCX 21 kb) [file 12864_2019_5851_MOESM6_ESM.docx]

**Additional file 6**

Estimated type-1 error rates with simulation data based on simulated RNA-seq data from negative binomial distribution. Estimated type-1 error rates by BALLI, DESeq2, edgeR, LLI, and voom and their 95% confidence levels were estimated for $N=4, 6, 8, 28, 40, 64, and 68$. The type-1 error rates are marked by bold font if their 95% confidence levels include or lower than the nominal significant level $\alpha$.

| $\alpha$ | *N =* 4 | | | | | *N* = 6 | | | | |
| --- | --- | --- | --- | --- | --- | --- | --- | --- | --- | --- |
|  | BALLI | DESeq2 | edgeR | LLI | voom | BALLI | DESeq2 | edgeR | LLI | voom |
| 0.1 | 0.16520  (0.16357,  0.16683) | **0.05832**  **(0.05707,**  **0.05958)** | 0.10380  (0.10118,  0.10642) | 0.26460  (0.26275,  0.26645) | **0.09865**  **(0.09634,**  **0.10096)** | 0.11493  (0.11314,  0.11672) | **0.07286**  **(0.07178,**  **0.07395)** | 0.10501  (0.10315,  0.10687) | 0.17998  (0.17789,  0.18208) | **0.09748**  **(0.09612,**  **0.09883)** |
| 0.05 | 0.09943  (0.09802,  0.10084) | **0.02826**  **(0.02762,**  **0.02889)** | 0.05170  (0.05050,  0.05289) | 0.18810  (0.18639,  0.18981) | **0.03975**  **(0.03878,**  **0.04072)** | 0.06526  (0.06400,  0.06652) | **0.03619**  **(0.03544,**  **0.03695)** | 0.05191  (0.05103,  0.05280) | 0.11408  (0.11231,  0.11585) | **0.04854**  **(0.04741,**  **0.04967)** |
| 0.01 | 0.01911  (0.01849,  0.01974) | **0.00598**  **(0.00568,**  **0.00627)** | 0.01160  (0.01112,  0.01208) | 0.08166  (0.08031,  0.08300) | **0.00315**  **(0.00279,**  **0.00352)** | 0.01719  (0.01663,  0.01776) | **0.00840**  **(0.00791,**  **0.00890)** | 0.01196  (0.01131,  0.01260) | 0.04234  (0.04138,  0.04331) | **0.00989**  **(0.00930,**  **0.01047)** |
| 0.005 | 0.00798  (0.00761,  0.00836) | **0.00304**  **(0.00272,**  **0.00335)** | 0.00615  (0.00575,  0.00654) | 0.05304  (0.05205,  0.05403) | **0.00087**  **(0.00072,**  **0.00103)** | 0.00845  (0.00805,  0.00886) | **0.00454**  **(0.00419,**  **0.00488)** | 0.00653  (0.00610,  0.00696) | 0.02672  (0.02596,  0.02749) | **0.00501**  **(0.00471,**  **0.00531)** |
| $\alpha$ | *N* = 8 | | | | | *N* = 28 | | | | |
|  | BALLI | DESeq2 | edgeR | LLI | voom | BALLI | DESeq2 | edgeR | LLI | voom |
| 0.1 | 0.10329  (0.10216,  0.10443) | **0.07871**  **(0.07731,**  **0.08012)** | 0.10720  (0.10565,  0.10875) | 0.14907  (0.14759,  0.15056) | **0.10068**  **(0.09925,**  **0.10211)** | **0.09328**  **(0.09212,**  **0.09444)** | **0.08584**  **(0.08462,**  **0.08705)** | 0.11761  (0.11631,  0.11890) | 0.10702  (0.10561,  0.10842) | **0.10019**  **(0.09896,**  **0.10142)** |
| 0.05 | 0.05334  (0.05255,  0.05414) | **0.04026**  **(0.03946,**  **0.04107)** | 0.05303  (0.05198,  0.05409) | 0.09150  (0.09051,  0.09249) | **0.04990**  **(0.04902,**  **0.05079)** | **0.04538**  **(0.04433,**  **0.04643)** | **0.04319**  **(0.04224,**  **0.04415)** | 0.06144  (0.06032,  0.06255) | 0.05491  (0.05371,  0.05611) | **0.05042**  **(0.04951,**  **0.05133)** |
| 0.01 | 0.01293  (0.01240,  0.01345) | **0.00935**  **(0.00888,**  **0.00981)** | 0.01216  (0.01176,  0.01256) | 0.02780  (0.02706,  0.02854) | 0.01073  (0.01018,  0.01127) | **0.00847**  **(0.00806,**  **0.00888)** | **0.00976**  **(0.00932,**  **0.01020)** | 0.01305  (0.01259,  0.01351) | 0.01168  (0.01120,  0.01216) | **0.01021**  **(0.00975,**  **0.01067)** |
| 0.005 | 0.00731  (0.00698,  0.00763) | **0.00522**  **(0.00488,**  **0.00555)** | 0.00668  (0.00634,  0.00702) | 0.01754  (0.01701,  0.01806) | 0.00556  (0.00519,  0.00593) | **0.00419**  **(0.00396,**  **0.00442)** | **0.00521**  **(0.00492,**  **0.00550)** | 0.00703  (0.00672,  0.00733) | 0.00592  (0.00554,  0.00630) | **0.00499**  **(0.00464,**  **0.00534)** |
| $\alpha$ | *N =* 40 | | | | | *N* = 64 | | | | |
|  | BALLI | DESeq2 | edgeR | LLI | voom | BALLI | DESeq2 | edgeR | LLI | voom |
| 0.1 | **0.09283**  **(0.09136,**  **0.09429)** | **0.08589**  **(0.08480,**  **0.08698)** | 0.11000  (0.10879,  0.11121) | 0.10280  (0.10136,  0.10424) | **0.10117**  **(0.09977,**  **0.10257)** | **0.09326**  **(0.09185,**  **0.09467)** | **0.08703**  **(0.08574,**  **0.08831)** | 0.10597  (0.10447,  0.10748) | **0.09916**  **(0.09777,**  **0.10055)** | **0.10041**  **(0.09903,**  **0.10178)** |
| 0.05 | **0.04455**  **(0.04352,**  **0.04557)** | **0.04335**  **(0.04228,**  **0.04442)** | 0.05918  (0.05799,  0.06038) | 0.05156  (0.05040,  0.05272) | **0.05007**  **(0.04905,**  **0.05110)** | **0.04591**  **(0.04518,**  **0.04663)** | **0.04431**  **(0.04332,**  **0.04530)** | 0.05501  (0.05388,  0.05614) | **0.05010**  **(0.04942,**  **0.05078)** | **0.05017**  **(0.04927,**  **0.05107)** |
| 0.01 | **0.00855**  **(0.00812,**  **0.00897)** | **0.00933**  **(0.00893,**  **0.00973)** | 0.01278  (0.01217,  0.01338) | 0.01062  (0.01018,  0.01106) | **0.00990**  **(0.00934,**  **0.01045)** | **0.00875**  **(0.00830,**  **0.00921)** | **0.00955**  **(0.00909,**  **0.0100)** | 0.01294  (0.01238,  0.01350) | **0.01011**  **(0.00954,**  **0.01069)** | **0.01001**  **(0.00952,**  **0.01050)** |
| 0.005 | **0.00426**  **(0.00391,**  **0.00460)** | **0.00496**  **(0.00464,**  **0.00529)** | 0.00678  (0.00639,  0.00717) | 0.00547  (0.00511,  0.00582) | **0.00520**  **(0.00487,**  **0.00553)** | **0.00430**  **(0.00397,**  **0.00462)** | **0.00499**  **(0.00465,**  **0.00533)** | 0.00705  (0.00659,  0.00752) | **0.00508**  **(0.00477,**  **0.00540)** | **0.00496**  **(0.00462,**  **0.00531)** |
| $\alpha$ | *N* = 68 | | | | |  |  |  |  |  |
|  | BALLI | DESeq2 | edgeR | LLI | voom |  |  |  |  |  |
| 0.1 | **0.09295**  **(0.09143,**  **0.09447)** | **0.08675**  **(0.08533,**  **0.08817)** | 0.10526  (0.10372,  0.10679) | **0.09834**  **(0.09685,**  **0.09984)** | **0.09967**  **(0.09821,**  **0.10112)** |  |  |  |  |  |
| 0.05 | **0.04541**  **(0.04445,**  **0.04637)** | **0.04382**  **(0.04295,**  **0.04468)** | 0.05415  (0.05313,  0.05518) | **0.04936**  **(0.04831,**  **0.05040)** | **0.04999**  **(0.04909,**  **0.05089)** |  |  |  |  |  |
| 0.01 | **0.00856**  **(0.00814,**  **0.00898)** | **0.00944**  **(0.00895,**  **0.00992)** | 0.01258  (0.01197,  0.01319) | **0.00984**  **(0.00935,**  **0.01033)** | **0.00992**  **(0.00938,**  **0.01045)** |  |  |  |  |  |
| 0.005 | **0.00443**  **(0.00402,**  **0.00485)** | **0.00502**  **(0.00466,**  **0.00538)** | 0.00681  (0.00638,  0.00725) | **0.00507**  **(0.00462,**  **0.00552)** | **0.00513**  **(0.00474,**  **0.00551)** |  |  |  |  |  |
