# Supplementary material for: BALLI: Bartlett-adjusted likelihood-based linear model approach for identifying differentially expressed genes with RNA-seq data
Source: BMC Genomics. 2019 Jul 2;20:540. doi: 10.1186/s12864-019-5851-6 (PMC6604381; doi:10.1186/s12864-019-5851-6)
Supplement: Supplementary file 7 — Effect of varying library sizes on the type-1 error rates when u = 0.2, 0.4, 0.6, 0.8, and 1 and N = 12, 16, 20, 24, 28, 40, 64, or 68 at the 0.005 nominal significance level. (DOCX 371 kb) [file 12864_2019_5851_MOESM7_ESM.docx]

**Additional file 7**

Effect of varying library sizes on the type-1 error rates. Type-1 error rates were estimated by BALLI, DESeq2, edgeR, LLI, and voom when u = 0.2, 0.4, 0.6, 0.8, and 1 and sample size (N) is 12, 16, 20, 24, 28, 40, 64, or 68 at the 0.005 nominal significance level. When *N* = 12, estimated values of LLI are out of bound, where the exact values of the rates are 0.00983, 0.01057, 0.01016, 0.01035, and 0.01043 at u = 0.2, 0.4, 0.6, 0.8, and 1, respectively.

**
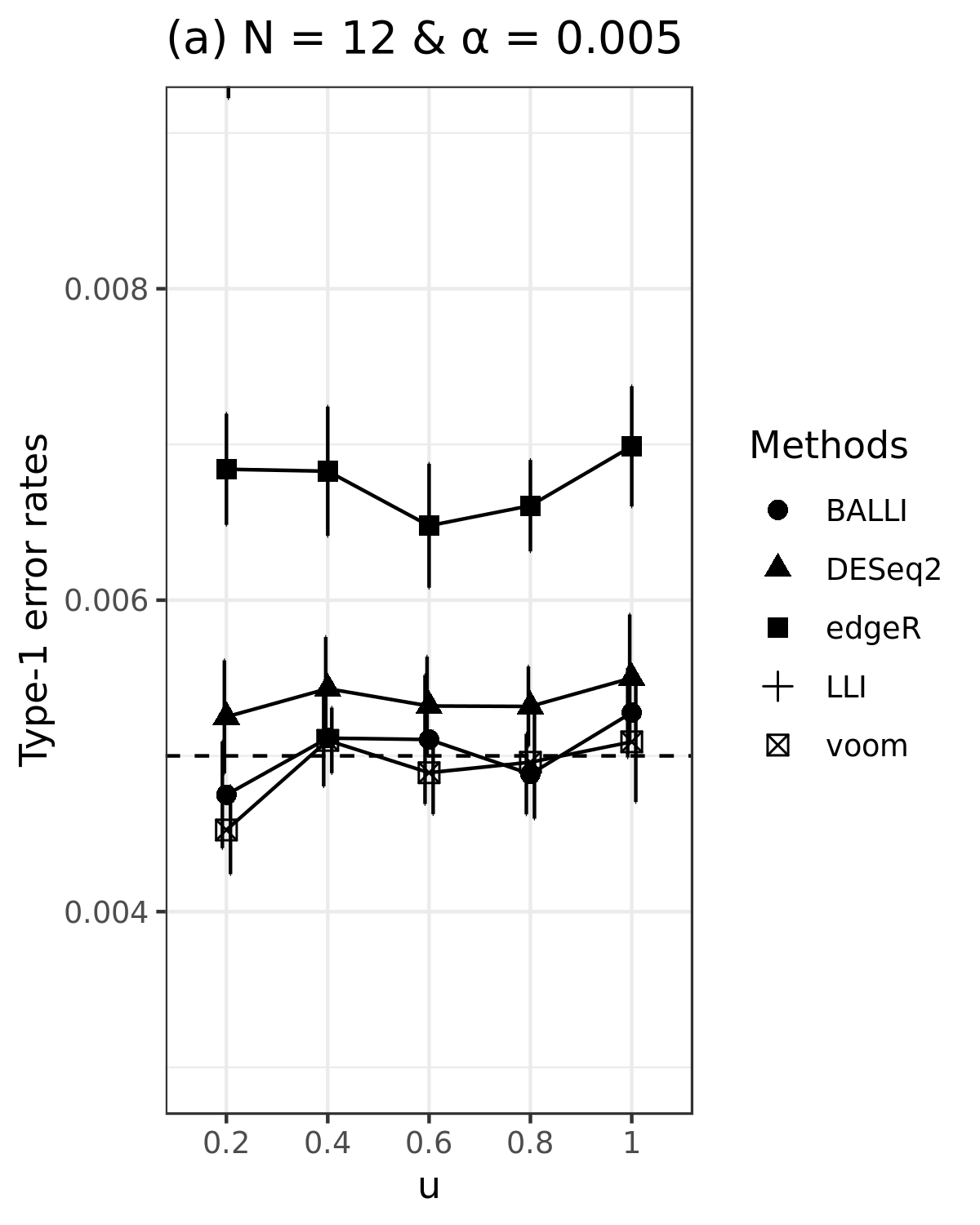

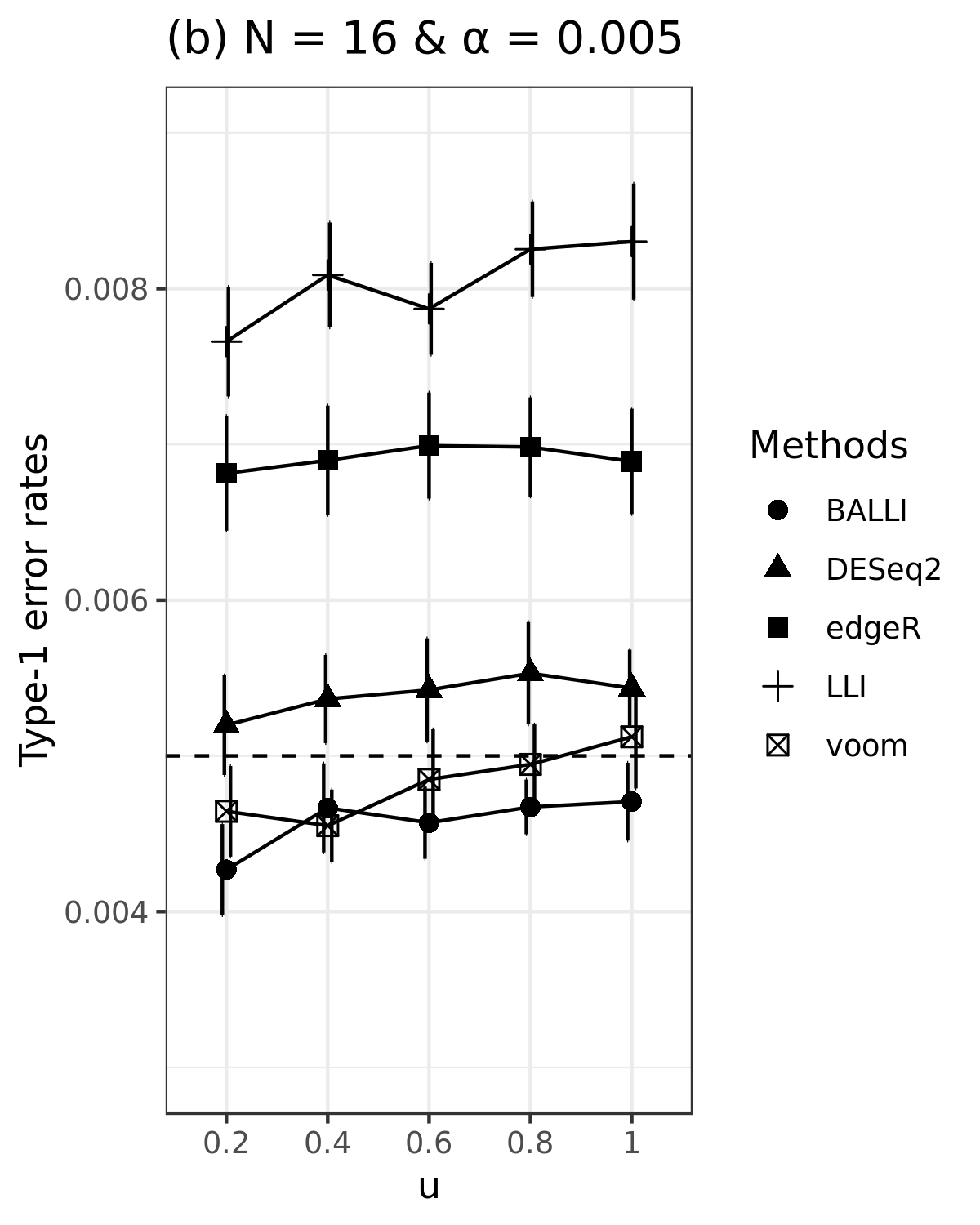

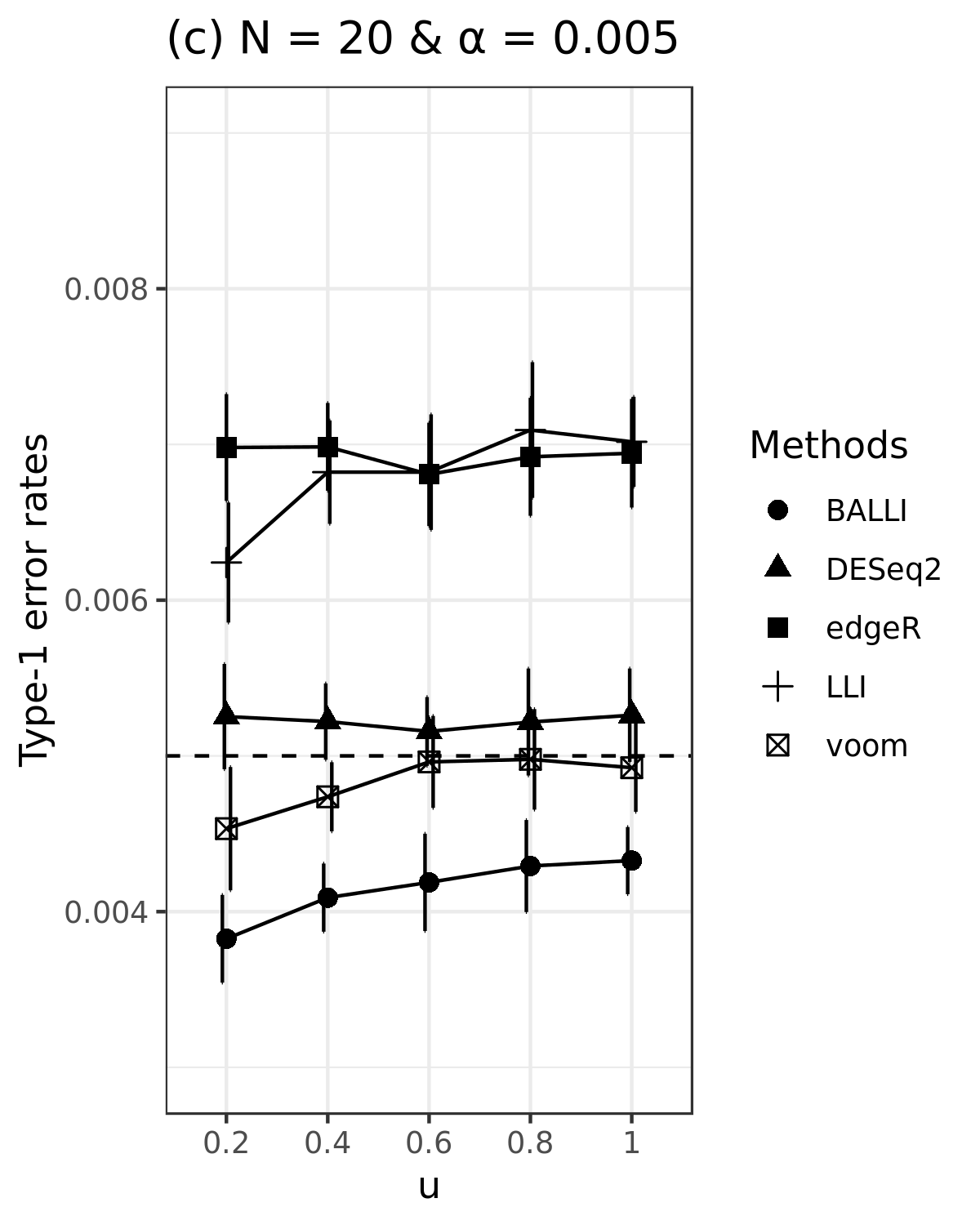

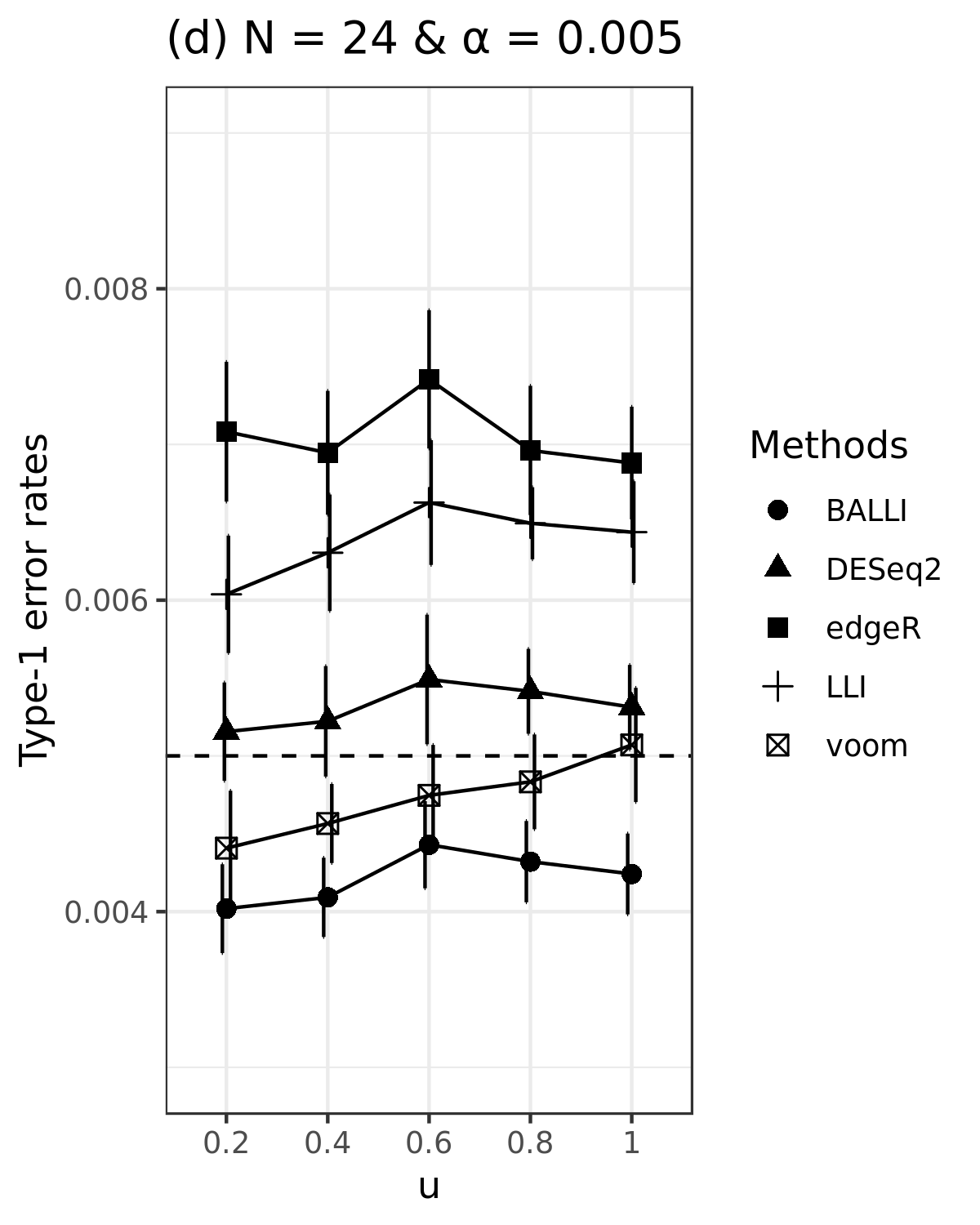

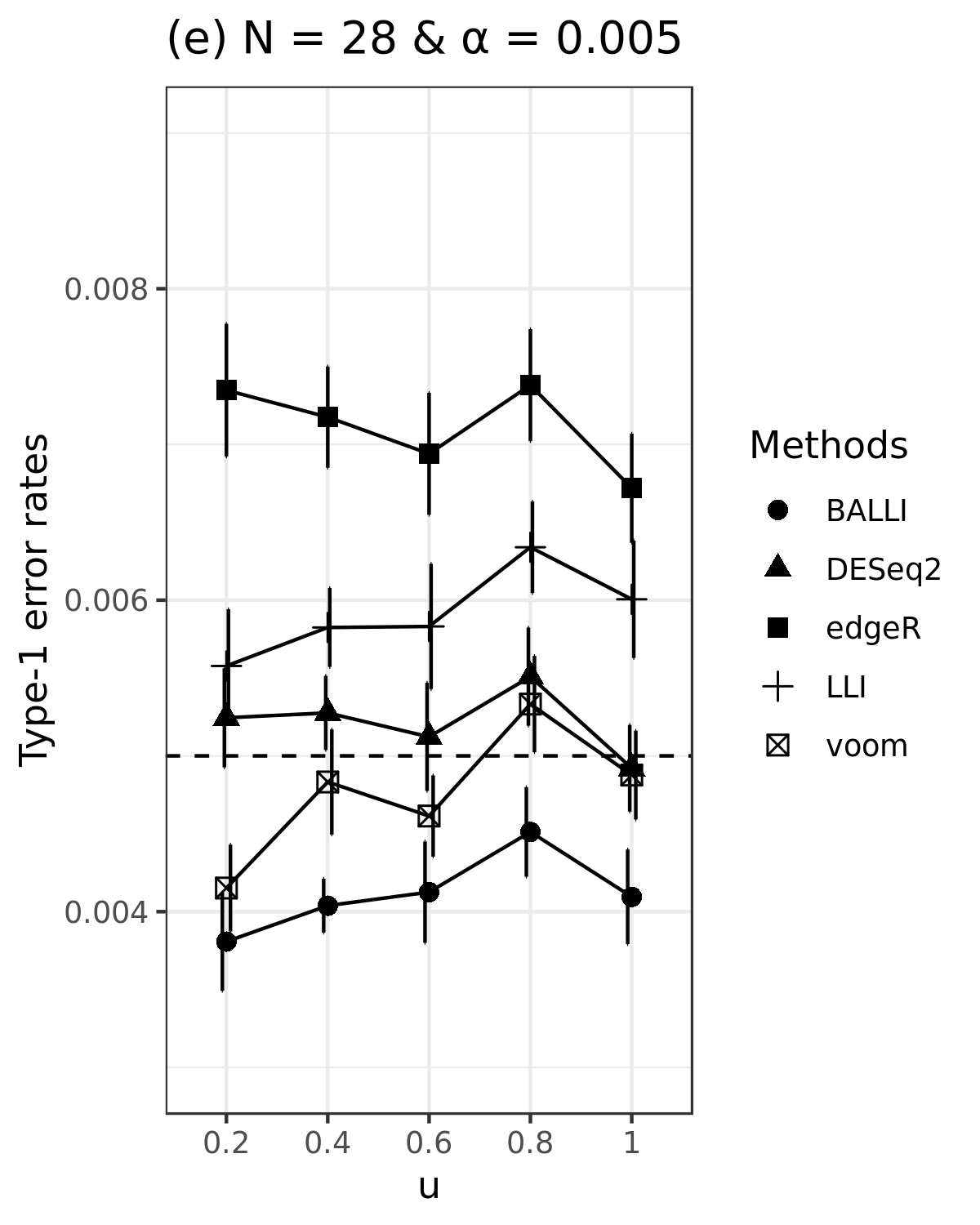

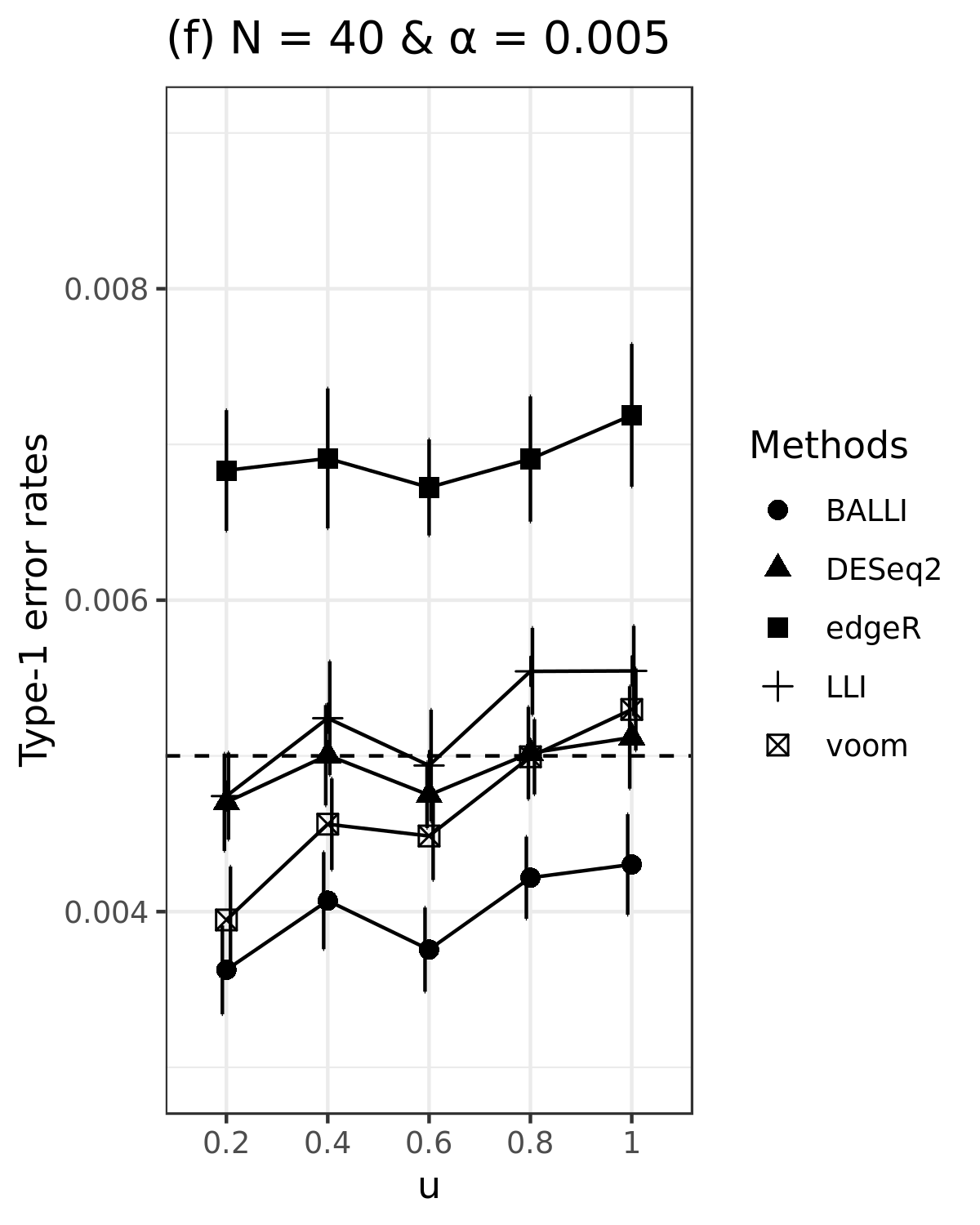

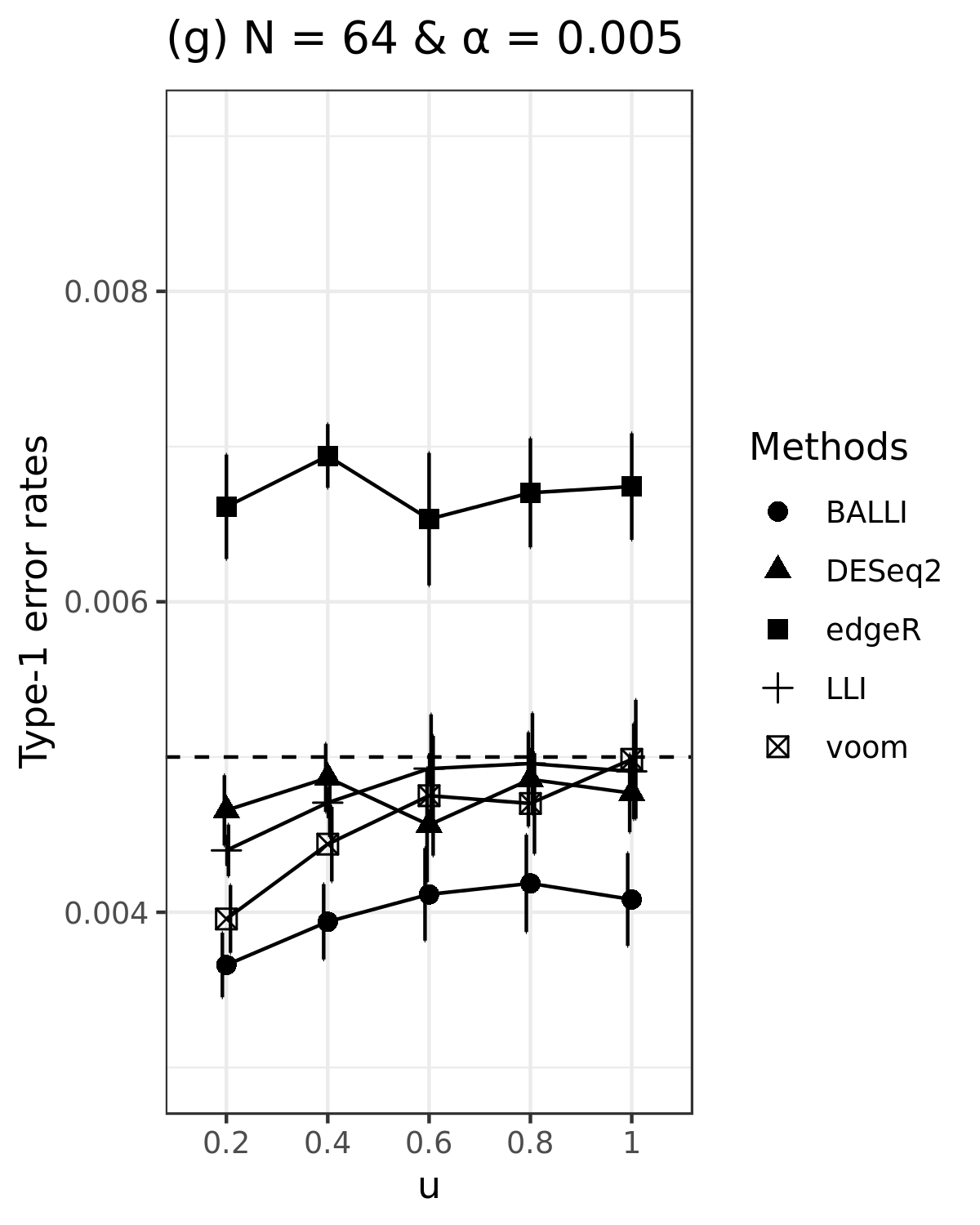

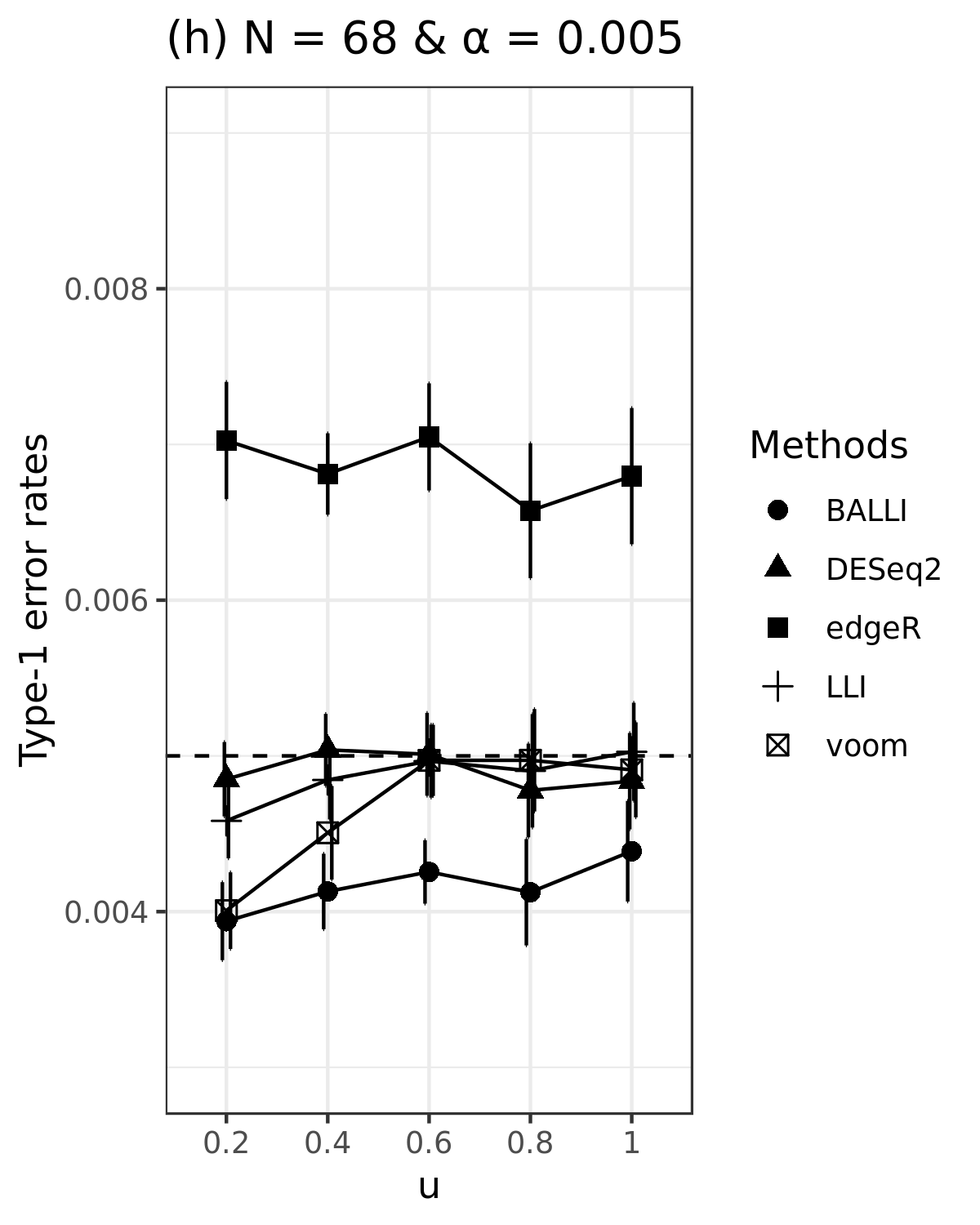
**
