# Supplementary material for: BALLI: Bartlett-adjusted likelihood-based linear model approach for identifying differentially expressed genes with RNA-seq data
Source: BMC Genomics. 2019 Jul 2;20:540. doi: 10.1186/s12864-019-5851-6 (PMC6604381; doi:10.1186/s12864-019-5851-6)

**Additional file 8**

Effect of varying library sizes on the statistical power and precision. Statistical powers and precisions for BALLI, DESeq2, edgeR, LLI, and voom were empirically estimated at FDR-adjusted 0.1 significance level when u = 1, 0.8, 0.6, or 0.4, δ = 1σ and sample size (N) is 12, 16, 20, 24, 28, 40, 64, or 68.


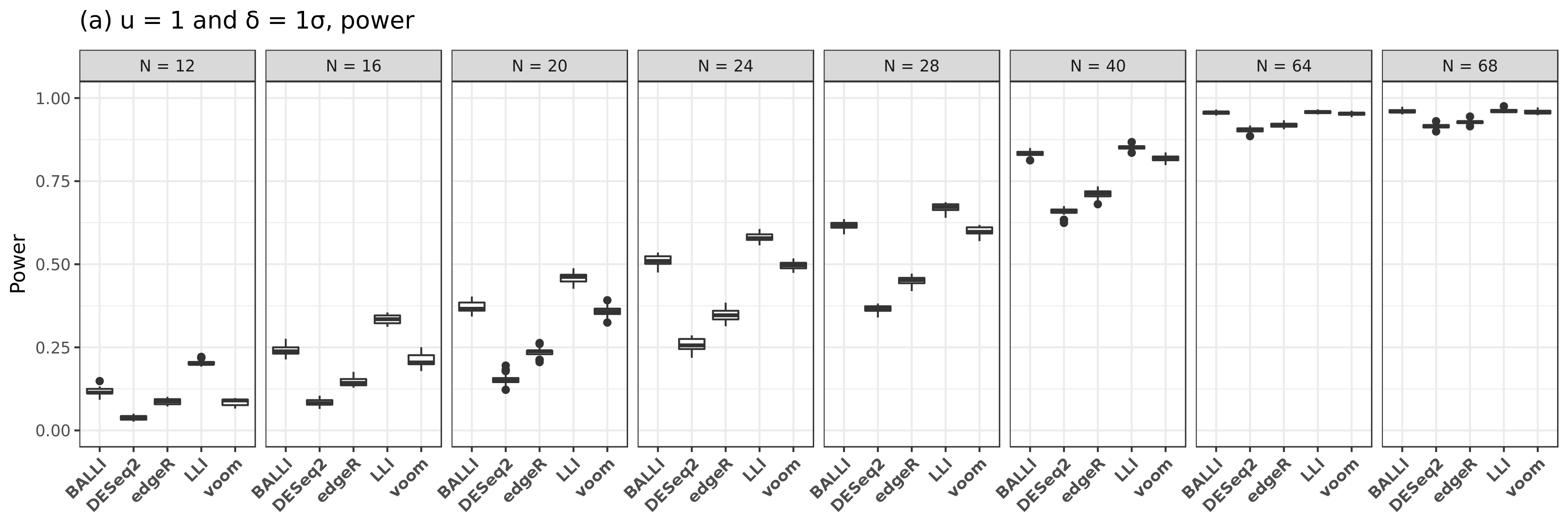

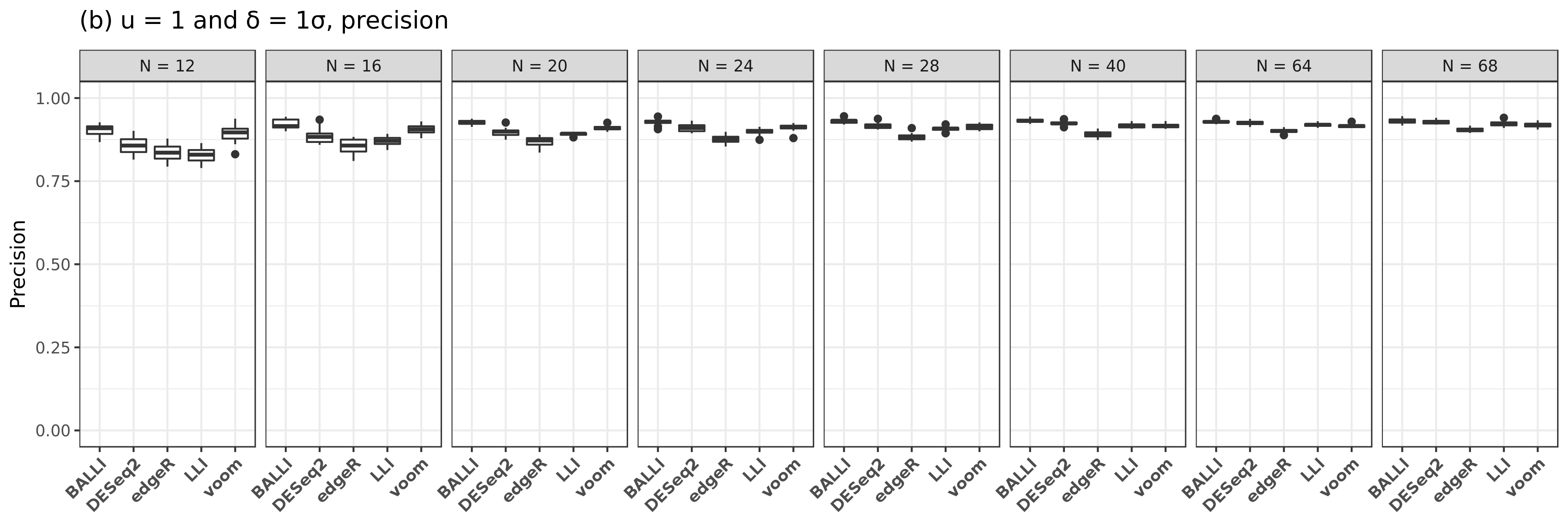

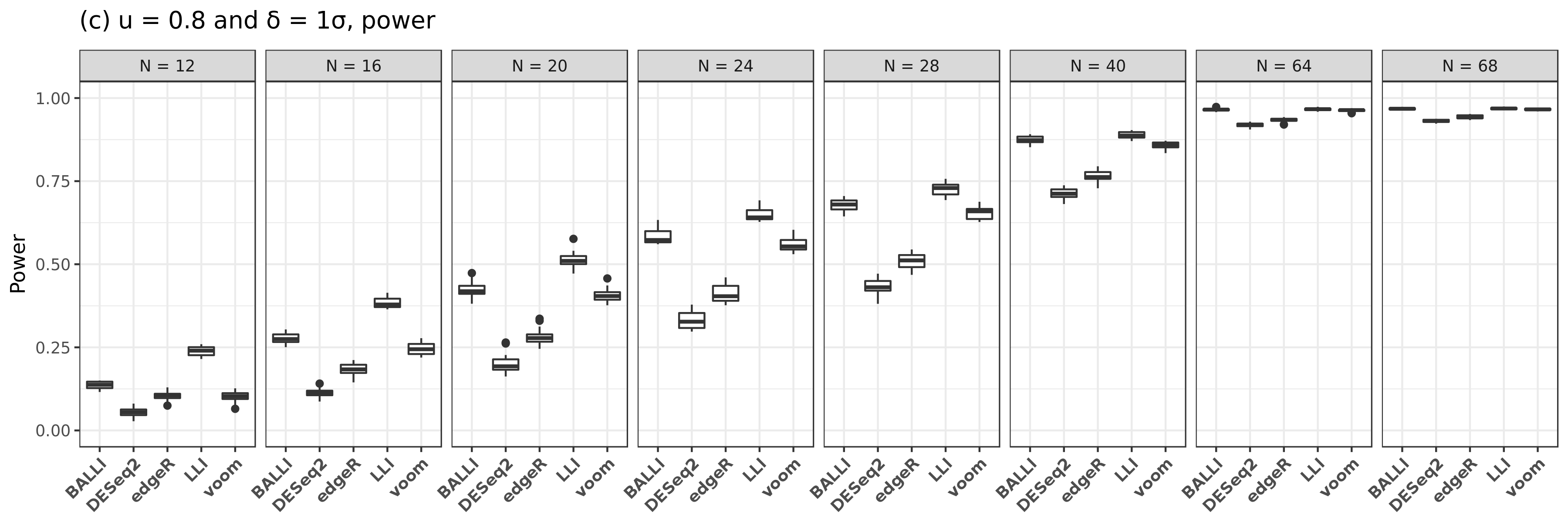

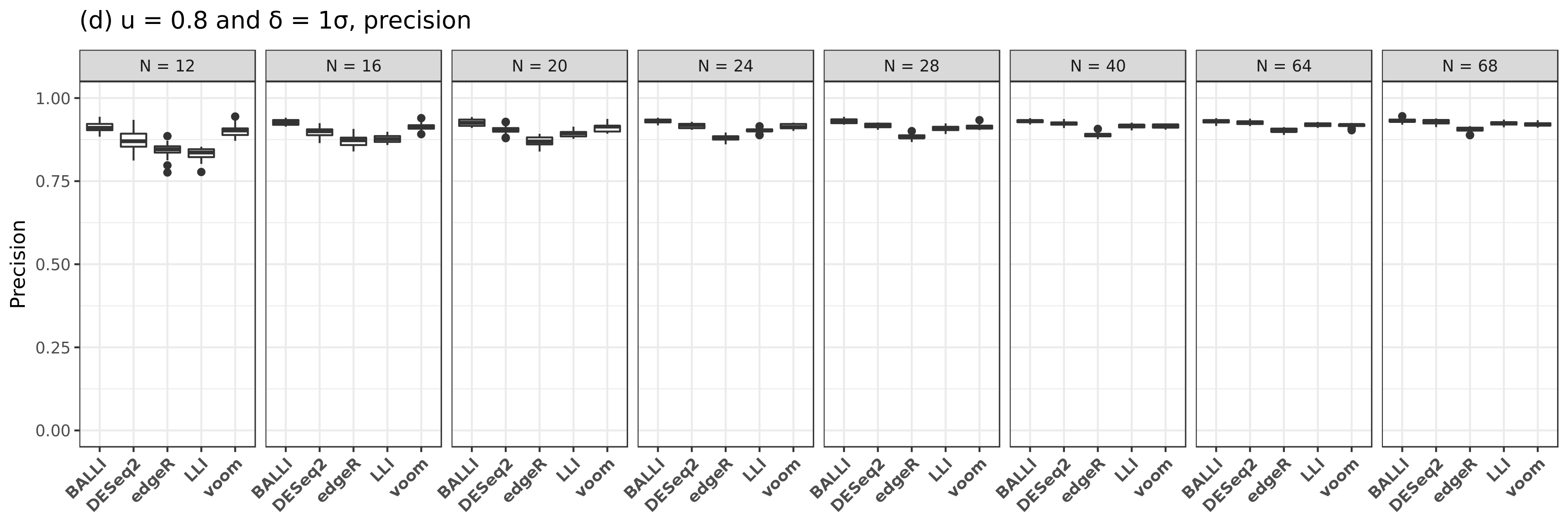

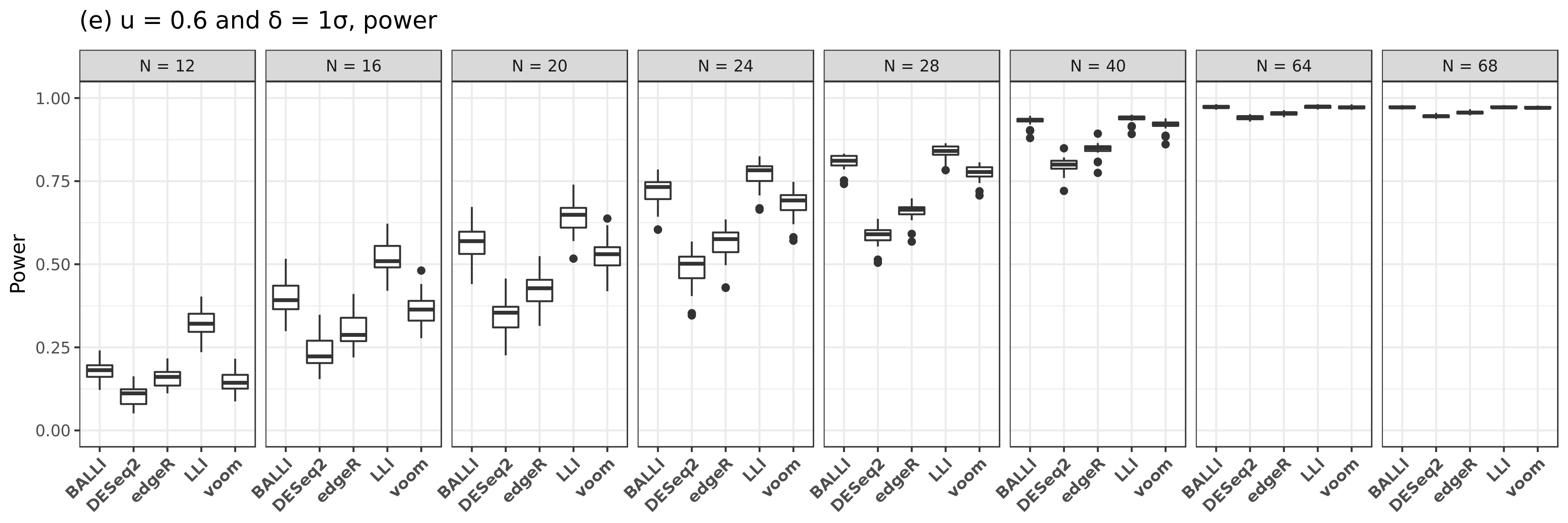

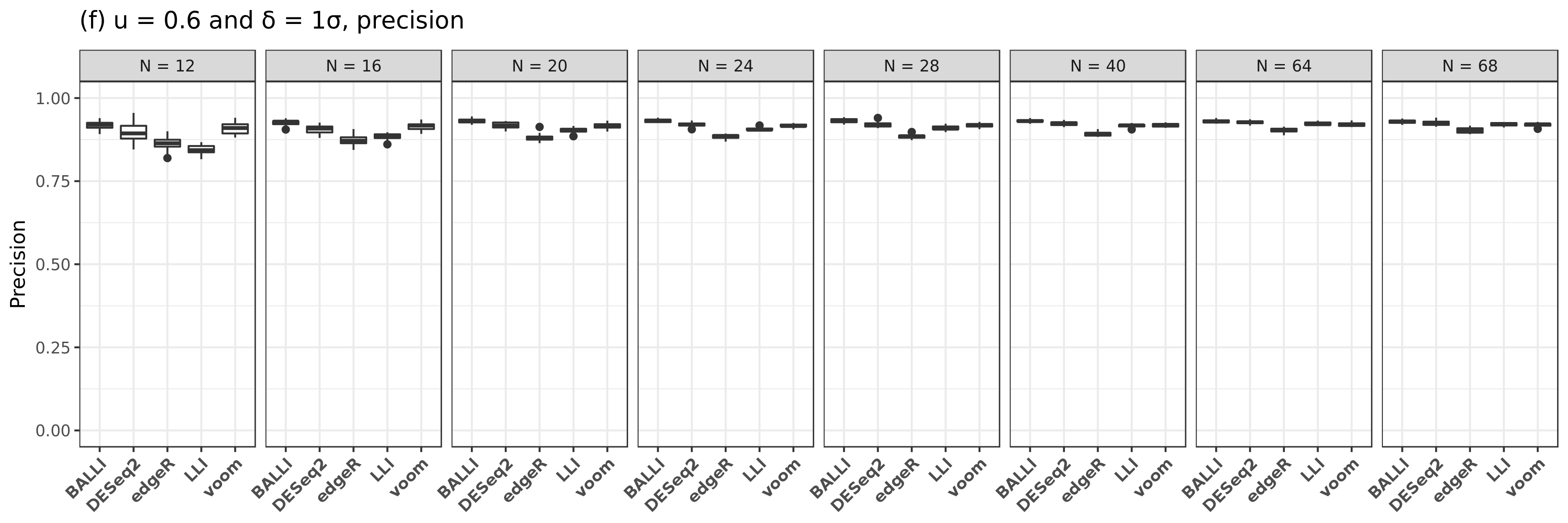

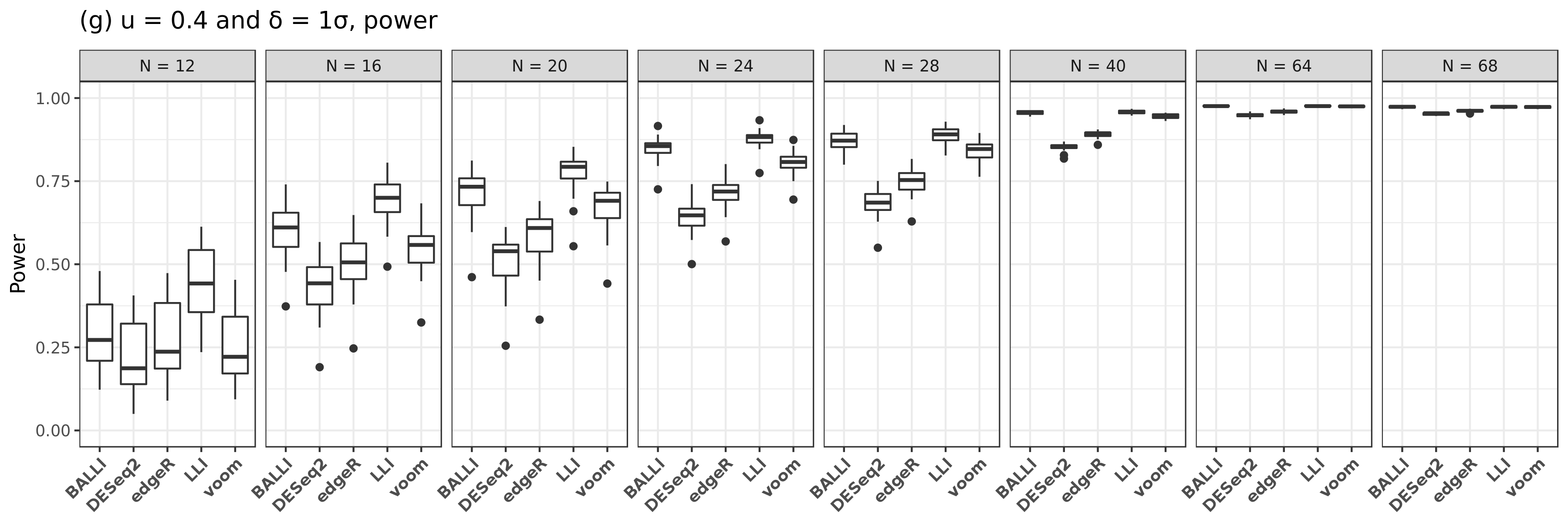

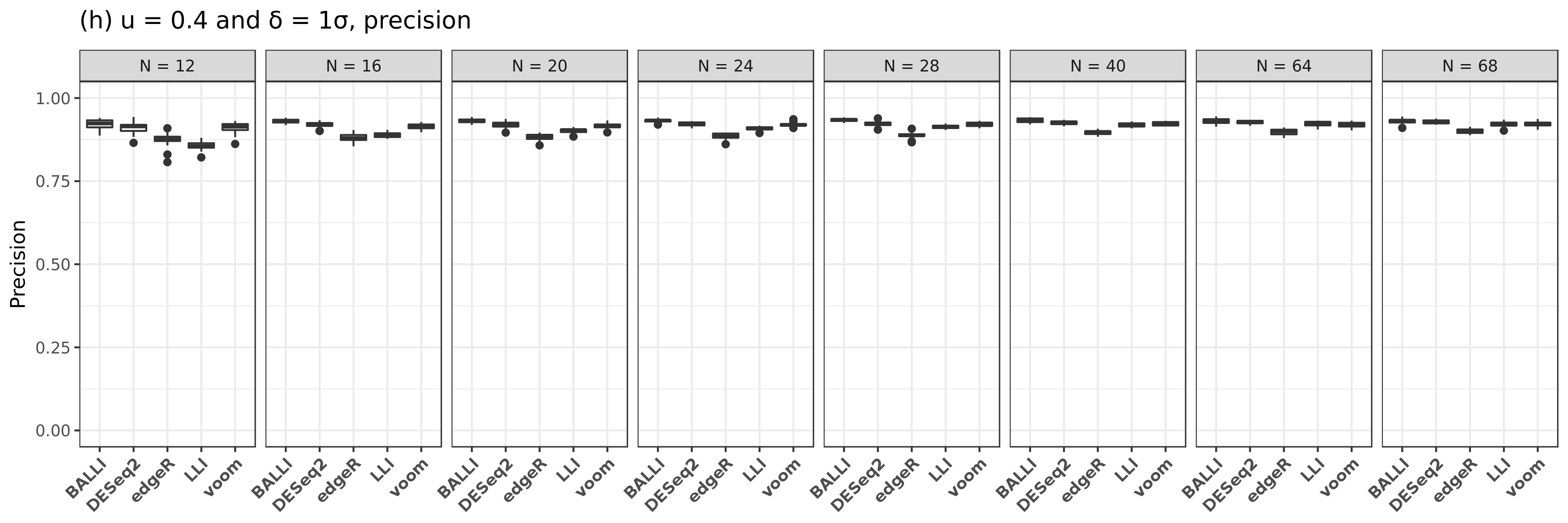

Supplement: Supplementary file 8 — Effect of varying library sizes on the statistical power and precision when u = 1, 0.8, 0.6, or 0.4, δ = 1σ and N = 12, 16, 20, 24, 28, 40, 64, or 68. (DOCX 593 kb) [file 12864_2019_5851_MOESM8_ESM.docx]
