# Supplementary material for: BALLI: Bartlett-adjusted likelihood-based linear model approach for identifying differentially expressed genes with RNA-seq data
Source: BMC Genomics. 2019 Jul 2;20:540. doi: 10.1186/s12864-019-5851-6 (PMC6604381; doi:10.1186/s12864-019-5851-6)
Supplement: Supplementary file 9 — Analysis of covariables, parity and lactation period, for true nine DEGs of Holstein cow's data. (DOCX 18 kb) [file 12864_2019_5851_MOESM9_ESM.docx]

**Additional file 9**

Analysis of covariables, parity and lactation period, for true nine DEGs of Holstein milk data. Holstein milk data was analyzed by BALLI, DESeq2, edgeR, LLI, and voom and their p values (FDRs) are provided. Because parity is categorical variable which has the value of 2, 3 or 4, LRT option was used for DESeq2 instead of default Wald option and voom cannot be applied.

|  | Parity | | | | |
| --- | --- | --- | --- | --- | --- |
|  | BALLI | DESeq2 | edgeR | LLI | voom |
| TOX4 | 2.542×10^-1^  (1) | 5.632×10^-1^  (1) | 8.380×10^-1^  (1) | 1.893×10^-1^  (8.850×10^-1^) | - |
| HNRNPL | 1.983×10^-1^  (1) | 6.994×10^-1^  (1) | 8.370×10^-1^  (1) | 1.402×10^-1^  (8.773×10^-1^) | - |
| SPTSSB | 9.158×10^-1^  (1) | 8.901×10^-1^  (1) | 8.005×10^-1^  (1) | 8.977×10^-1^  (9.856×10^-1^) | - |
| NOS3 | 4.956×10^-1^  (1) | 4.194×10^-1^  (1) | 4.435×10^-1^  (1) | 4.239×10^-1^  (9.526×10^-1^) | - |
| SLC4A1 | 2.451×10^-1^  (1) | 3.969×10^-1^  (1) | 4.120×10^-1^  (1) | 1.775×10^-1^  (8.850×10^-1^) | - |
| NLN | 8.068×10^-1^  (1) | 5.392×10^-1^  (1) | 6.752×10^-1^  (1) | 7.703×10^-1^  (9.728×10^-1^) | - |
| KALRN | 3.594×10^-1^  (1) | 5.872×10^-1^  (1) | 6.095×10^-1^  (1) | 2.876×10^-1^  (9.214×10^-1^) | - |
| PMCH | 9.411×10^-1^  (1) | 6.325×10^-1^  (1) | 6.615×10^-1^  (1) | 9.289×10^-1^  (9.913×10^-1^) | - |
| C25H16orf88 | 4.125×10^-1^  (1) | 6.578×10^-1^  (1) | 6.867×10^-1^  (1) | 3.411×10^-1^  (9.400×10^-1^) | - |

|  | Lactation Period | | | | |
| --- | --- | --- | --- | --- | --- |
|  | BALLI | DESeq2 | edgeR | LLI | voom |
| TOX4 | 9.804×10^-1^  (9.908×10^-1^) | 7.714×10^-1^  (9.274×10^-1^) | 9.684×10^-1^  (9.892×10^-1^) | 9.780×10^-1^  (9.884×10^-1^) | 9.839×10^-1^  (9.950×10^-1^) |
| HNRNPL | 3.387×10^-1^  (5.813×10^-1^) | 8.084×10^-1^  (9.396×10^-1^) | 7.454×10^-1^  (8.942×10^-1^) | 2.824×10^-1^  (4.845×10^-1^) | 6.463×10^-1^  (8.444×10^-1^) |
| SPTSSB | 1.086×10^-1^  (3.433×10^-1^) | 6.056×10^-1^  (8.359×10^-1^) | 5.280×10^-1^  (7.725×10^-1^) | 7.055×10^-2^  (2.245×10^-1^) | 3.184×10^-1^  (6.128×10^-1^) |
| NOS3 | 2.090×10^-1^  (4.570×10^-1^) | 3.604×10^-1^  (6.449×10^-1^) | 2.891×10^-1^  (5.979×10^-1^) | 1.562×10^-1^  (3.425×10^-1^) | 1.468×10^-1^  (4.403×10^-1^) |
| SLC4A1 | 7.833×10^-2^  (3.030×10^-1^) | 6.648×10^-2^  (2.756×10^-1^) | 4.941×10^-2^  (2.927×10^-1^) | 4.665×10^-2^  (1.836×10^-1^) | 5.084×10^-2^  (2.932×10^-1^) |
| NLN | 8.947×10^-1^  (9.509×10^-1^) | 7.587×10^-1^  (9.214×10^-1^) | 8.396×10^-1^  (9.380×10^-1^) | 8.817×10^-1^  (9.375×10^-1^) | 9.116×10^-1^  (9.678×10^-1^) |
| KALRN | 2.145×10^-1^  (4.623×10^-1^) | 5.654×10^-1^  (8.124×10^-1^) | 5.379×10^-1^  (7.784×10^-1^) | 1.627×10^-1^  (3.509×10^-1^) | 2.159×10^-1^  (5.167×10^-1^) |
| PMCH | 9.843×10^-1^  (9.925×10^-1^) | 6.261×10^-1^  (8.487×10^-1^) | 7.004×10^-1^  (8.724×10^-1^) | 9.823×10^-1^  (9.905×10^-1^) | 8.294×10^-1^  (9.367×10^-1^) |
| C25H16orf88 | 7.587×10^-2^  (3.012×10^-1^) | 8.526×10^-2^  (3.089×10^-1^) | 2.203×10^-1^  (5.353×10^-1^) | 4.610×10^-2^  (1.829×10^-1^) | 2.048×10^-1^  (5.070×10^-1^) |
