# Supplementary material for: BALLI: Bartlett-adjusted likelihood-based linear model approach for identifying differentially expressed genes with RNA-seq data
Source: BMC Genomics. 2019 Jul 2;20:540. doi: 10.1186/s12864-019-5851-6 (PMC6604381; doi:10.1186/s12864-019-5851-6)
Supplement: Supplementary file 10 — Computation time for each method when analyzing Holstein cow’s data. (DOCX 14 kb) [file 12864_2019_5851_MOESM10_ESM.docx]

**Additional file 10**

Computation time for each method when analyzing Holstein cow’s RNA-seq real data whose number of samples (N) is 21, number of genes (G) is 11,968 and number of variables (P) including intercept is 5. The analysis was performed on Intel Xeon E7-4820 2.00 GHz processor using a single core. For BALLI, result using 20 cores is represented in the parenthesis

| Method | Computation Time  (*N* = 21, *G* = 11,968 and *P* = 5) |
| --- | --- |
| BALLI | 153.35 sec (11.99 sec) |
| DESeq2 | 27.21 sec |
| edgeR | 33.25 sec |
| voom | 2.81 sec |
